# A Genome-Based Model to Predict the Virulence of Pseudomonas aeruginosa Isolates

**DOI:** 10.1128/mBio.01527-20

**Published:** 2020-08-25

**Authors:** Nathan B. Pincus, Egon A. Ozer, Jonathan P. Allen, Marcus Nguyen, James J. Davis, Deborah R. Winter, Chih-Hsien Chuang, Cheng-Hsun Chiu, Laura Zamorano, Antonio Oliver, Alan R. Hauser

**Affiliations:** a Department of Microbiology-Immunology, Northwestern University Feinberg School of Medicine, Chicago, Illinois, USA; b Department of Medicine, Division of Infectious Diseases, Northwestern University Feinberg School of Medicine, Chicago, Illinois, USA; c University of Chicago Consortium for Advanced Science and Engineering, University of Chicago, Chicago, Illinois, USA; d Division of Data Science and Learning, Argonne National Laboratory, Argonne, Illinois, USA; e Northwestern-Argonne Institute of Science and Engineering, Evanston, Illinois, USA; f Department of Medicine, Division of Rheumatology, Northwestern University Feinberg School of Medicine, Chicago, Illinois, USA; g School of Medicine, College of Medicine, Fu-Jen Catholic University, New Taipei, Taiwan; h Molecular Infectious Disease Research Center, Chang Gung Memorial Hospital, Chang Gung University, Taoyuan, Taiwan; i Servicio de Microbiología y Unidad de Investigación, Hospital Universitari Son Espases, Institut d’Investigació Sanitaria Illes Balears, Palma de Mallorca, Spain; Georgia Institute of Technology School of Biological Sciences

**Keywords:** *Pseudomonas aeruginosa*, genome analysis, machine learning, modeling, prediction, virulence

## Abstract

Variation in the genome of Pseudomonas aeruginosa, an important pathogen, can have dramatic impacts on the bacterium’s ability to cause disease. We therefore asked whether it was possible to predict the virulence of P. aeruginosa isolates based on their genomic content. We applied a machine learning approach to a genetically and phenotypically diverse collection of 115 clinical P. aeruginosa isolates using genomic information and corresponding virulence phenotypes in a mouse model of bacteremia. We defined the accessory genome of these isolates through the presence or absence of accessory genomic elements (AGEs), sequences present in some strains but not others. Machine learning models trained using AGEs were predictive of virulence, with a mean nested cross-validation accuracy of 75% using the random forest algorithm. However, individual AGEs did not have a large influence on the algorithm’s performance, suggesting instead that virulence predictions are derived from a diffuse genomic signature. These results were validated with an independent test set of 25 P. aeruginosa isolates whose virulence was predicted with 72% accuracy. Machine learning models trained using core genome single-nucleotide variants and whole-genome k-mers also predicted virulence. Our findings are a proof of concept for the use of bacterial genomes to predict pathogenicity in P. aeruginosa and highlight the potential of this approach for predicting patient outcomes.

## Abstract (precis)

Pseudomonas aeruginosa is a clinically important Gram-negative opportunistic pathogen. P. aeruginosa shows a large degree of genomic heterogeneity both through variation in sequences found throughout the species (core genome) and through the presence or absence of sequences in different isolates (accessory genome). P. aeruginosa isolates also differ markedly in their ability to cause disease. In this study, we used machine learning to predict the virulence level of P. aeruginosa isolates in a mouse bacteremia model based on genomic content. We show that both the accessory and core genomes are predictive of virulence. This study provides a machine learning framework to investigate relationships between bacterial genomes and complex phenotypes such as virulence.

## INTRODUCTION

Pseudomonas aeruginosa is a ubiquitous Gram-negative opportunistic pathogen that infects a variety of hosts. Its ability to cause severe acute infections in susceptible patients and chronic infections in individuals with cystic fibrosis, coupled with increasing rates of antimicrobial resistance, make it an organism of particular concern to the medical community (1–3). The P. aeruginosa species, however, is not monolithic. Instead, it shows a large degree of genomic diversity both through polymorphisms and differences in gene content (4–6). As routine whole-genome sequencing becomes increasingly feasible, understanding how these genomic differences impact the pathogenicity of P. aeruginosa may allow clinicians to rapidly identify high-risk infections and researchers to select the most high-yield strains for further study.

As with other bacteria, the genome of P. aeruginosa can be divided into a core genome, made up of sequences common to the species, and an accessory genome, made up of sequences present in some strains but not others (6, 7). While only 10 to 15% of a typical strain’s genome is accessory, when combined from all strains these sequences comprise the vast majority of the P. aeruginosa pangenome (4, 7, 8). Variations in both the core and accessory genomes impact the virulence of any given P. aeruginosa strain. Core genome mutations that accumulate in P. aeruginosa strains during chronic infection of cystic fibrosis patients lead to decreased *in vitro* virulence markers (9), and these strains have attenuated virulence in animal models of acute infection (10). Genomic islands, major components of the accessory genome, are enriched for predicted virulence factors (11). Several genomic islands in P. aeruginosa, including those containing the type III secretion system (T3SS) effector gene *exoU*, have been shown to enhance pathogenicity in multiple infection models (12–14). We recently identified, within the accessory genome, multiple novel virulence determinants in a mouse model of bacteremia (15). Conversely, a study using a Caenorhabditis elegans model identified several P. aeruginosa accessory genes whose presence reduced virulence (16). Furthermore, the presence of active CRISPR systems was associated with increased virulence (16), supporting the hypothesis that many horizontally transferred elements are genetic parasites with respect to the host bacterium (17). Because of its role in both increasing and decreasing the pathogenicity of individual P. aeruginosa strains, the accessory genome may serve as a useful predictor of an isolate’s virulence. This prediction, however, is not as simple as detecting individual virulence or antivirulence factors. For example, *exoU* is a recognized virulence factor whose disruption dramatically attenuates a strain’s ability to cause disease (18, 19), but some strains naturally lacking *exoU* are more virulent than those possessing the gene (15). As virulence is a complex and combinatorial phenotype, the strategy taken to study it must be appropriately robust to that complexity.

In supervised machine learning, training samples that belong to known classes are used to build a computational model that can then predict the class of new samples (20). Supervised machine learning is an increasingly important tool in bacterial genomics and has been extensively applied to the prediction of antimicrobial resistance and identification of potential resistance determinants. This approach has proven successful in a variety of species and using a variety of genomic features (21–27). These studies benefited from readily available whole-genome sequencing and resistance data, as well as from an often easily explainable phenotype. Researchers have also begun to apply machine learning techniques to predict bacterial pathogenicity. Examples include using discriminatory single-nucleotide variants (SNVs) to predict Staphylococcus aureus *in vitro* cytotoxicity (28), using variation in core genome loci to predict patient mortality in specific S. aureus clones (29) and using predicted perturbations in protein coding sequences to classify *Salmonella* strains as causing either gastrointestinal or extraintestinal infections (30). A support vector machine approach has been used to distinguish the transcriptomes of P. aeruginosa in human infection compared to those in *in vitro* growth (31). However, to our knowledge there has been no study directly modeling P. aeruginosa pathogenicity from genomic content.

In this study, we utilize a supervised machine learning approach to predict P. aeruginosa virulence in a mouse model of bloodstream infection based on genomic content. We found that there is a signal within the accessory genome predictive of virulence, a finding validated using an independent test set of isolates. The predictions appear to be through the detection of a diffuse genetic fingerprint rather than individual virulence or antivirulence genes. The core genome also showed a predictive signal for virulence.

## RESULTS

### Genomic and virulence characterization of P. aeruginosa strains.

**FIG 1:**
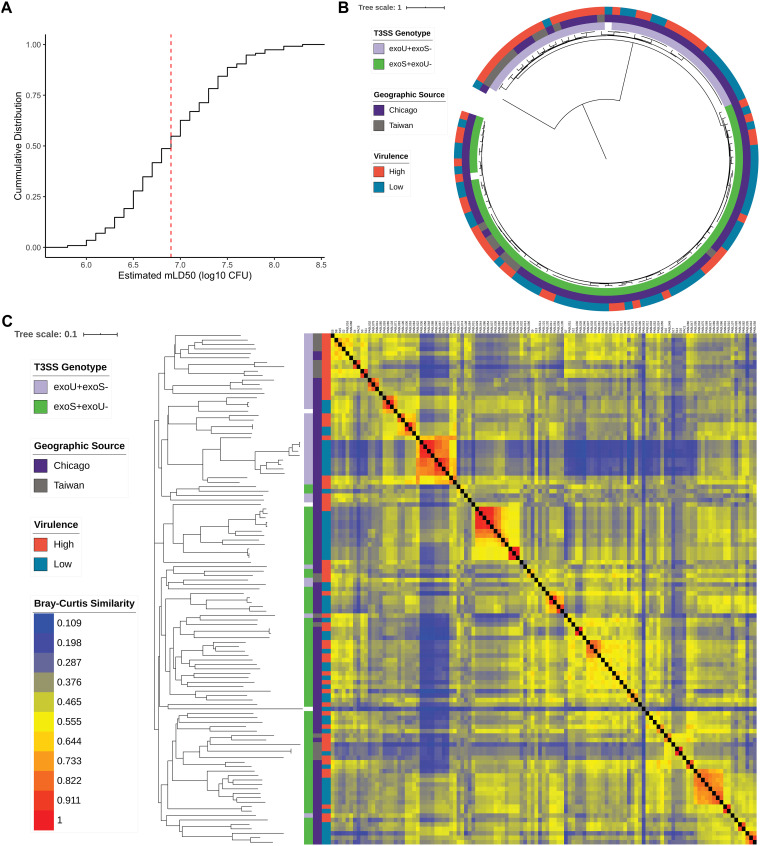
Virulence and genomic characteristics of the training set of 115 P. aeruginosa isolates. (A) Cumulative distribution function of estimated mLD_50_ values for the 115 isolates in a mouse model of bacteremia. Isolates with estimated mLD_50_ values less than the median value (red dashed line) were designated high virulence, with the remainder designated low virulence. (B) Midpoint rooted core genome phylogenetic tree of the 115 training isolates constructed from SNV loci present in at least 95% of genomes, annotated with T3SS genotype, geographic source, and virulence level. (C) Bray-Curtis dissimilarity heatmap comparing AGE presence in the 115 training isolates, weighted by AGE length, and accompanying neighbor joining tree. Isolates are annotated (from left to right) by T3SS genotype, geographic source, virulence level, and the dissimilarity heatmap. A higher value indicates that two isolates have more similar accessory genomes.

To assess whether the P. aeruginosa genome can be used to predict a given isolate’s virulence, we needed a large number of P. aeruginosa isolates with known whole-genome sequences and *in vivo* virulence data. We used two previously reported collections, 98 archived isolates from adults with bacteremia at Northwestern Memorial Hospital (NMH) in Chicago, IL, USA (32), and 17 isolates from children with Shanghai fever, a P. aeruginosa infection presenting with sepsis and gastrointestinal symptoms, at Chang Gung Children’s Hospital in Taiwan (33) (see Table S1 in the supplemental material). These 115 isolates formed our training set. We performed whole-genome sequencing for each of the isolates that had not been previously sequenced. Likewise, we supplemented previously reported virulence data (15, 33) with additional experiments (see Table S2 in the supplemental material) to approximate the CFU of each bacterial isolate necessary to cause prelethal illness in 50% of mice using a bacteremia model. From these data, we estimated a modified 50% lethal dose (mLD_50_) for each of the 115 P. aeruginosa isolates (see Table S3 in the supplemental material). The isolates showed a median mLD_50_ of 6.9 log_10_ CFU but a wide range of pathogenicity in mice, differing by over 100-fold in the dose required to cause severe disease, as was previously reported for the NMH isolates (15). For the purpose of this study, we classified isolates with an estimated mLD_50_ below the median value for the group as “high virulence” and the remainder as “low virulence” (Fig. 1A). These results provided a large collection of P. aeruginosa isolates with known whole-genome sequences and virulence in a mouse bacteremia model.

10.1128/mBio.01527-20.5 TABLE S1 Pseudomonas aeruginosa isolates included in this study. Download Table S1, XLSX file, 0.02 MB. Copyright © 2020 Pincus et al. 2020 Pincus et al. This content is distributed under the terms of the Creative Commons Attribution 4.0 International license.

10.1128/mBio.01527-20.6 TABLE S2 Mouse survival in a tail-vein model of bacteremia for isolates included in this study. Download Table S2, XLSX file, 0.02 MB. Copyright © 2020 Pincus et al. 2020 Pincus et al. This content is distributed under the terms of the Creative Commons Attribution 4.0 International license.

10.1128/mBio.01527-20.7 TABLE S3 Estimated 50% lethal dose (LD_50_) values for isolates included in this study. Download Table S3, XLSX file, 0.01 MB. Copyright © 2020 Pincus et al. 2020 Pincus et al. This content is distributed under the terms of the Creative Commons Attribution 4.0 International license.

We performed a phylogenomic analysis to assess the diversity of the core genomes of all 115 isolates in the training set (Fig. 1B). The core genome phylogenetic tree showed that the isolates are largely nonclonal and were found in both major clades of the species, which are mainly differentiable by the near-mutually exclusive presence of the T3SS effector genes *exoS* or *exoU* (4, 5). One distinct outlier isolate from the PA7-like clade was also present in the collection (4). The *exoU^+^* clade contained a larger proportion of highly virulent isolates than the *exoS*^+^ clade. Although some clusters of closely related isolates shared the same virulence class, both major clades contained high- and low-virulence isolates.

We next defined the accessory genome of each of the 115 isolates in the training set. The accessory genome can be divided into accessory genomic elements (AGEs), discrete sequences found in the genomes of some isolates but not others (7). For the purpose of this study, noncontiguous accessory sequences were grouped and considered a single AGE if they were perfectly correlated (present and absent from the same isolates in the training set). Sets of accessory sequences totaling less than 200 bp were excluded from analysis. Using this approach, a total of 3,013 AGEs, with a mean length of 4,059 bp, a median length of 672 bp, and forming a pan-accessory genome of 12.2 Mb, were identified in these isolates (see Table S4 in the supplemental material). A Bray-Curtis dissimilarity heatmap of AGE presence/absence, weighted by the length of each AGE, shows that there is considerable accessory genomic variability in our collection (Fig. 1C). Consistent with previous findings (4), the clade containing *exoS* and the clade containing *exoU* largely separate based on accessory genomic content, as evidenced by both Bray-Curtis dissimilarity and multiple correspondence analysis. Similar to the core genome phylogenetic analysis, some clusters of isolates with similar accessory genomes share a virulence rank, but both high- and low-virulence isolates show diverse AGE content (Fig. 1C and Fig. S1A and B).

10.1128/mBio.01527-20.1 FIG S1 Multiple correspondence analysis (MCA) of accessory genomic content. MCA was performed based on AGE presence/absence in the 115 training set isolates and annotated based on (A) type III secretion system (T3SS) genotype and (B) virulence level. MCA was also performed on all 140 isolates in both the training and test sets, considering only the 3,013 AGEs defined from the training set and annotated based on (C) T3SS genotype, (D) virulence level, and (E) dataset. The first two dimensions, and the percentage of variance they explain, are shown. Download FIG S1, TIF file, 1.7 MB. Copyright © 2020 Pincus et al. 2020 Pincus et al. This content is distributed under the terms of the Creative Commons Attribution 4.0 International license.

10.1128/mBio.01527-20.8 TABLE S4 Accessory genomic elements (perfectly correlated subelements of ≥200 bp) in the 115 training isolates. Download Table S4, XLSX file, 0.1 MB. Copyright © 2020 Pincus et al. 2020 Pincus et al. This content is distributed under the terms of the Creative Commons Attribution 4.0 International license.

### Evaluating machine learning models predicting P. aeruginosa virulence based on accessory genome content.

We hypothesized that, as the P. aeruginosa accessory genome is variable between strains (6, 7, 34) and includes multiple known virulence determinants (12, 13, 15), it would contain information predictive of strain virulence in mice. To test this hypothesis, we took a supervised machine learning approach (see Fig. S2 in the supplemental material). Through this approach, we tested the performance of the following four commonly used machine learning algorithms: random forest, l2-regularized logistic regression, elastic net logistic regression, and support vector classifier. Accessory genome content, in the form of AGE presence/absence, was used as features, and virulence level (high or low) was used as labels during modeling. During model construction, optimal hyperparameters were chosen using grid search cross-validation. Here, all possible combinations of hyperparameters were tested through 10-fold cross-validation. The best-performing combination was then used to build a final model. Model performance was estimated using 10-fold nested cross-validation. In this process, grid search cross-validation was performed within an outer cross-validation loop. For each training fold in this outer loop, a model was built through grid search cross-validation, and its performance was tested against the cross-validation fold. Nested cross-validation does not return a final machine learning model but instead examines how multiple models perform against held-out data. This process provides an estimate of how well a model trained through a given strategy will generalize to new data.

10.1128/mBio.01527-20.2 FIG S2 Overview of the machine learning pipeline. AGE: accessory genomic element, CV: cross-validation. Download FIG S2, TIF file, 1 MB. Copyright © 2020 Pincus et al. 2020 Pincus et al. This content is distributed under the terms of the Creative Commons Attribution 4.0 International license.

**FIG 2:**
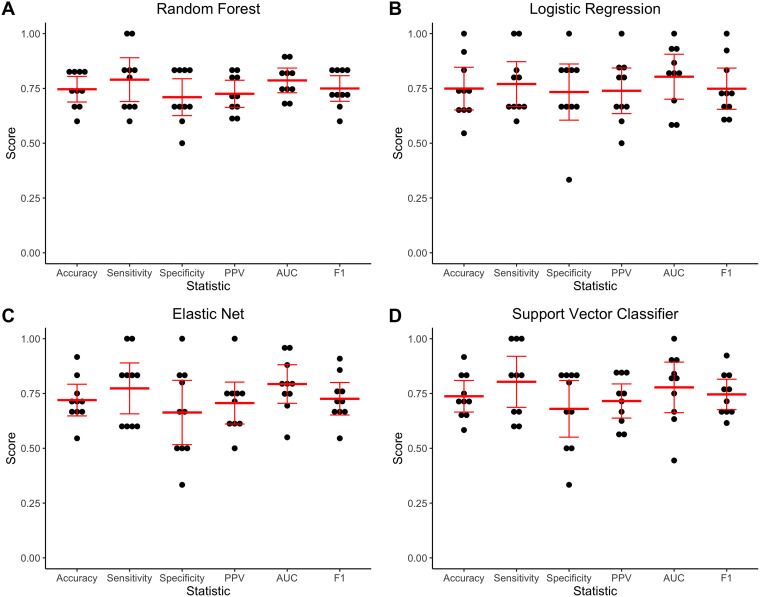
Nested 10-fold cross-validation performance of machine learning algorithms in predicting P. aeruginosa virulence in mice based on accessory genomic content. (A) Random forest, (B) l2-regularized logistic regression, (C) elastic net logistic regression, and (D) support vector classifier algorithms were tested. Accuracy, sensitivity, specificity, positive predictive value (PPV), area under the receiver operating characteristic curve (AUC), and F1 score were determined for each cross-validation fold (black dots). The mean and 95% confidence interval of each statistic are indicated in red.

All four algorithms performed similarly, with mean nested cross-validation accuracies of 0.75 (95% confidence interval [95% CI], 0.69 to 0.80) for random forest, 0.75 (95% CI, 0.65 to 0.85) for l2-regularized logistic regression, 0.72 (95% CI, 0.65 to 0.79) for elastic net logistic regression, and 0.74 (95% CI, 0.67 to 0.81) for support vector classifier. Other performance metrics showed similar ranges of values (Fig. 2). Notably, the accuracy of all four algorithms was substantially higher than the null accuracy of simply predicting all isolates to be the majority class, which in this case was the prevalence of low-virulence isolates (0.51). This indicates that there is signal in the accessory genome predictive of virulence in P. aeruginosa. Since all four machine learning algorithms performed similarly in nested cross-validation, we chose the random forest approach for further investigation.

**FIG 3:**
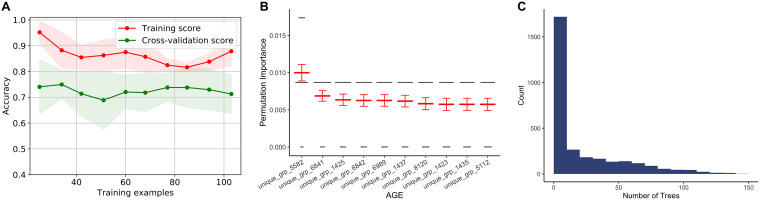
Evaluation of the random forest algorithm in predicting P. aeruginosa virulence based on accessory genomic content. (A) Learning curve showing change in mean training accuracy (red line) and cross-validation accuracy (green line) in predicting P. aeruginosa virulence as increasing numbers of isolates are used to train the random forest model. Shading indicates the 95% confidence interval. Assessments at each number of training examples were through 10-fold nested cross-validation. (B) Out-of-bag permutation importance for the 10 most important AGEs in the random forest model, showing decrease in accuracy when these AGEs were randomly permuted. Permutation importance testing was performed 100 times, with the results of each test represented by the width of the black lines and the mean and 95% confidence interval indicated in red for each AGE. (C) Histogram indicating how many trees within the random forest model contained each AGE (feature), out of a total of 10,000 trees.

We next evaluated whether sample size limited the performance of the random forest approach. We tested how accuracy of a model changed with increasing training set size, both against training and cross-validation examples (Fig. 3A). While the training and cross-validation performance for the random forest model did not completely converge as more training examples were added, the learning curve showed that we are unlikely to see substantial improvement in cross-validation accuracy with additional training isolates. A caveat to this result is that the learning curve can only consider AGEs contained in the training set and cannot account for the impact of additional AGEs (or different patterns of AGE carriage) found when including new genetically distinct isolates.

To further probe the characteristics of the random forest approach, we built a final random forest model using all 115 isolates in the training set. The out-of-bag accuracy (performance on the out-of-bag samples not included in each of the 10,000 decision trees making up the random forest) of this model was 0.75 (see Table S5 in the supplemental material), which is consistent with our nested cross-validation results. When assessed against the training isolates, the model showed an accuracy of 0.79, consistent with the trend in training accuracies observed in the learning curve (Table S5 and Fig. 3A). The training accuracy can be thought of as an idealized maximal performance and supports the conclusion that additional training examples are unlikely to substantially improve the model.

10.1128/mBio.01527-20.9 TABLE S5 Performance of the accessory genome random forest model against the training set of 115 P. aeruginosa isolates. Download Table S5, XLSX file, 0.01 MB. Copyright © 2020 Pincus et al. 2020 Pincus et al. This content is distributed under the terms of the Creative Commons Attribution 4.0 International license.

10.1128/mBio.01527-20.10 DATA SET S1 Sequences of subelements of >50 bp making up the 10 accessory genomic elements (AGEs) most predictive of virulence class in the random forest model trained on AGE content of the 115 training isolates. Download DATA SET S1, DOCX file, 0.03 MB. Copyright © 2020 Pincus et al. 2020 Pincus et al. This content is distributed under the terms of the Creative Commons Attribution 4.0 International license.

We next investigated which AGEs were most critical in making a prediction of high or low virulence in this model. We calculated the permutation importance (the mean decrease in model accuracy when a given feature is randomly permuted) for each AGE. To do this, we randomly permuted each AGE 100 times and then determined the impact on out-of-bag accuracy. Overall, individual features showed low importance in the predictions made by the model, with permutation of the most important AGE causing only a mean 1% drop in model accuracy (Fig. 3B). The vast majority of features (2,979/3,013) had no impact on out-of-bag accuracy when randomly permuted (Table S4), indicating that the machine learning model based decisions on a genomic signature predictive of virulence level rather than by identifying individual virulence or antivirulence factors. If a given AGE is randomly permuted, it appears that other correlated features compensate for it. Each individual AGE was included as a feature in a minority of the 10,000 decision trees, with the most prevalent AGE appearing in only 148 trees in the final model (Fig. 3C). As such, it was not possible for a single AGE to have a large impact on the prediction of virulence.

To further assess the apparent redundancy in our feature set, we randomly divided the 3,013 AGEs in the training set into 2, 4, and 10 subsets and evaluated the performance of random forest models built using only these subsets through nested cross-validation. We found that even when training on only a smaller subset of the accessory genomic features, model accuracy remained mostly unchanged (see Fig. S3A to C in the supplemental material). We next tested dividing the training AGEs into 100 random subsets and found that the average mean nested cross-validation accuracy across all subsets decreased to 0.67. Performance of many of the subsets deteriorated at this level of data reduction, with 14 subsets having a mean accuracy of <0.6, indicating that in some cases the remaining AGEs lacked sufficient signal to be good predictors of virulence (Fig. S3D). Together, these findings provide additional evidence that a broad genetic fingerprint, rather than individual virulence or antivirulence factors, is being used to classify strains as having high or low virulence. Furthermore, it is consistent with a recent finding that antimicrobial resistance in several species can be accurately predicted by only considering variation in a small subset of core genes (and excluding known resistance genes) (35).

10.1128/mBio.01527-20.3 FIG S3 Nested 10-fold cross-validation accuracy of a random forest model in predicting P. aeruginosa virulence when trained on random subsets of accessory genomic features. The 3,013 AGEs in the training set were randomly split into (A) 2, (B) 4, and (C) 10 subsets, and the accuracy of models trained using each of these subsets of features was estimated through nested cross-validation. The nested cross-validation accuracy obtained when all features are used for training (as in Fig. 2) is included for reference. For each subset, accuracy seen in each cross-validation fold are shown in black with the mean accuracy and 95% confidence interval indicated in red. The 3,013 AGEs in the training set were then split into (D) 100 subsets and the accuracy of models trained using each subset estimated through nested cross-validation. The mean nested cross-validation accuracy of each subset is shown in blue with the mean across all subsets indicated in red. Download FIG S3, TIF file, 1.9 MB. Copyright © 2020 Pincus et al. 2020 Pincus et al. This content is distributed under the terms of the Creative Commons Attribution 4.0 International license.

With the low permutation importance of any individual AGE, one must be cautious in drawing conclusions about their role in virulence. However, looking at the AGEs most predictive of virulence class and how they relate to one another may provide insights into genomic characteristics that are associated with, though not necessarily causative of, differences in pathogenicity. All of the 10 most predictive AGEs in the random forest model were more prevalent in low-virulence isolates (Table 1; see also Data File S1). Expanding this analysis to all AGEs with nonzero permutation importance showed that 32/34 were more prevalent in low virulence isolates (Table S4). This is consistent with the finding that horizontally acquired genetic elements, major components of the accessory genome (6, 17), can incur a fitness cost on the host bacterium (17). While some genomic islands encode virulence factors (11), many horizontally acquired elements can have a parasitic relationship with the bacterium (17). The AGE with the highest permutation importance aligns to a gene encoding the conjugative protein TraD, perhaps suggesting a general association of conjugative elements with reduced virulence. Four of the top 10 AGEs are comprised of sequences from the same “bin” in clustAGE analysis. This indicates that in at least some strains they are located near each other on the genome (i.e., part of a single, larger element). One of these four AGEs encodes an integrative and conjugative element (ICE) protein. These findings suggest that these AGEs are markers for a larger variable element common in low virulence strains. Two other AGEs are part of the same gene encoding a hypothetical protein. Finally, genes encoding arsenic resistance are highly prevalent in low-virulence isolates, perhaps suggesting either that this resistance comes at a cost or that strains adapted to survive heavy metal exposure are less able to cause disease in animals.

**TABLE 1:** AGEs most predictive of virulence in the accessory genome random forest model

AGE	Mean OOB permutation importance	Subelement(s)	Total length (bp)	Prevalence			Putative annotation^*a*^
				Total	High virulence	Low virulence	
unique_grp_5582	0.0100	bin364_se00006	433	0.417	0.161	0.661	TraD
unique_grp_6841	0.0069	bin610_se00004	902	0.304	0.107	0.492	Hypothetical protein
unique_grp_1425	0.0063	bin20_se00056	1717	0.330	0.125	0.525	TetR/AcrR family transcriptional regulator, short-chain dehydrogenase
unique_grp_6842	0.0063	bin610_se00005	369	0.296	0.089	0.492	Hypothetical protein
unique_grp_6989	0.0063	bin654_se00007	436	0.313	0.107	0.508	Intergenic region
unique_grp_1437	0.0062	bin20_se00073	2009	0.339	0.125	0.542	SoxR, MerR family DNA-binding transcriptional regulator, ICE relaxase PFGI-1 class, hypothetical protein
		bin20_se00075					
unique_grp_8120	0.0058	bin987_se00001	2821	0.339	0.125	0.542	AsrR family transcriptional regulators, arsenic transporter, arsenate reductase, ArsH, hypothetical protein
		bin1807_se00001					
unique_grp_1423	0.0057	bin20_se00054, bin20_se00057	1278	0.348	0.125	0.559	Type II glyceraldehyde-3-phosphate dehydrogenase
unique_grp_1435	0.0057	bin20_se00069	509	0.365	0.143	0.576	Hypothetical protein
unique_grp_5112	0.0057	bin258_se00005	419	0.357	0.143	0.559	ArsH

a Based on annotation of any open reading frame (ORF) with at least 50 bp overlap with the AGE sequence when a BLAST search was run against the *Pseudomonas* Genome Database (59).

### Assessing model performance with an independent test set.

**FIG 4:**
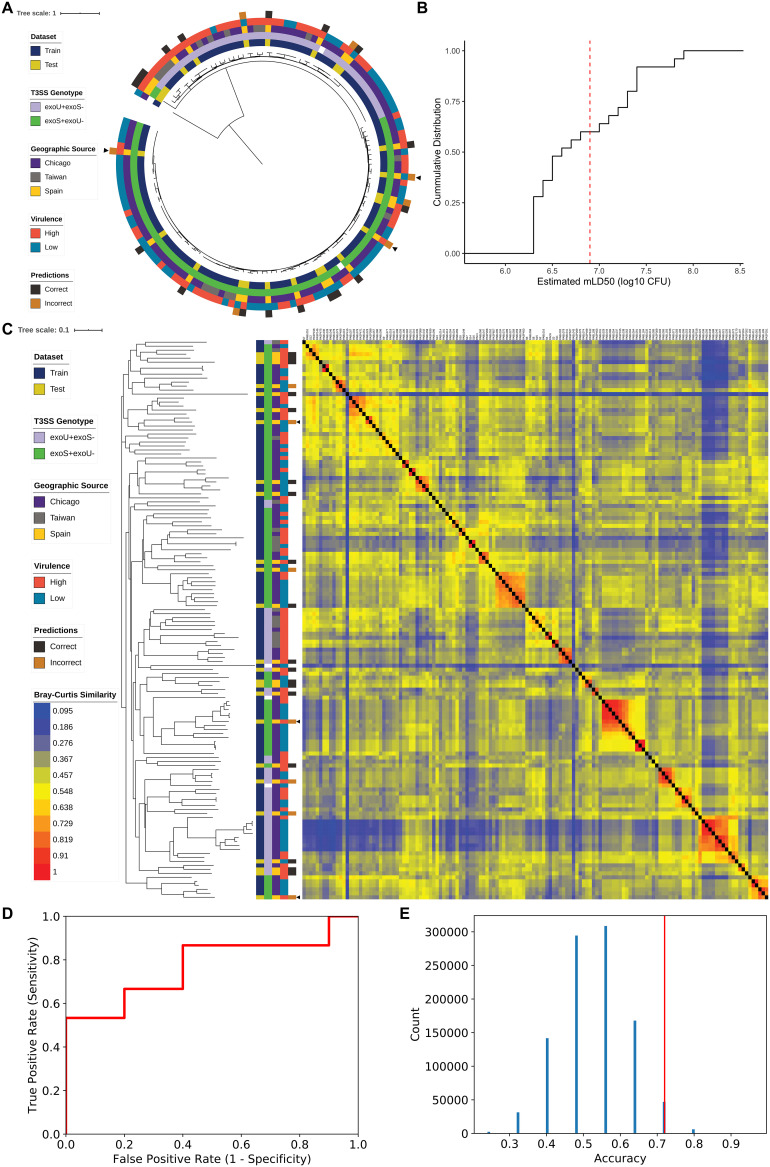
Characteristics of a random forest model trained on the accessory genomic content of the 115 P. aeruginosa training isolates to predict the virulence of an independent test set of 25 isolates. (A) Midpoint rooted core genome phylogenetic tree of the 115 training isolates and 25 test isolates constructed from SNV loci present in at least 95% of genomes, annotated (from inner to outer rings) with data set, T3SS genotype, geographic source, virulence level, and accuracy of prediction by the accessory genome random forest model for test set isolates. Arrowheads indicate examples of incorrectly classified test set strains whose closest core and accessory genomic neighbor(s) show a discordant virulence phenotype. (B) Cumulative distribution function of estimated mLD_50_ values for the 25 P. aeruginosa isolates making up the independent test set in a mouse model of bacteremia. Isolates with estimated mLD_50_ values less than the median estimated mLD_50_ of the training set (red dashed line) were designated high virulence, with the remainder designated low virulence. (C) Bray-Curtis dissimilarity heatmap comparing presence of the 3,013 AGEs identified in the training set in all 140 isolates, weighted by AGE length, and accompanying neighbor joining tree. Isolates are annotated (from left to right) by data set, T3SS genotype, geographic source, virulence level, accuracy of prediction by the accessory genome random forest model in test set isolates (arrowheads highlighting specific incorrectly classified test set strains as in panel A), and the dissimilarity heatmap. A higher value indicates that two isolates have more similar accessory genomes. (D) Receiver operating characteristic curve for predictions of the 25 test set isolates using the random forest model (AUC = 0.77). (E) Permutation analysis showing the likelihood of predicting test virulence with an accuracy of at least 0.72 if no true link between virulence and accessory genomic content existed. The predicted virulence of the 25 test isolates were randomly permuted 1 million times, and the resulting null distribution of possible model accuracies is shown. The vertical red line indicates the true accuracy of the random forest model in predicting test set virulence (one-sided *P* = 0.053).

The nested cross-validation performance of our random forest model provided an estimate of how well it would generalize to new P. aeruginosa isolates. To follow up on this, we applied the final random forest model built using all 115 training isolates to an independent test set of P. aeruginosa isolates to examine how well it predicted their virulence. As our test set, we selected 25 genetically diverse P. aeruginosa isolates previously cultured from patients with bacteremia in Spain between 2008 and 2009 (36) and for which we had whole-genome sequenced (Table S1 and Fig. 4A). The virulence of each isolate was assessed in the mouse model of bacteremia, and isolates were classified as high or low virulence using the same threshold (estimated mLD_50_ of 6.9 log_10_ CFU) defined for the training set (Fig. 4B and Tables S2 and S3 in the supplemental material). The test set was more pathogenic on average than the training set, with 15/25 (60%) of isolates classified as high virulence. This means that a trivial model uniformly predicting high virulence would show an accuracy of 0.6, higher than the null accuracy (0.51) of the training set. However, as the model we are testing was trained on a data set in which low virulence is the majority class (prevalence, 0.51), we would not expect this to occur. We identified which of the 3,013 AGEs used as training features were present in each of the test isolates. Adding these isolates to a Bray-Curtis dissimilarity heatmap of AGE presence/absence showed that the test set is also relatively diverse in accessory genomic content (Fig. 4C), a finding supported by multiple correspondence analysis (Fig. S1C to E).

We used the random forest model built with the training set accessory genomic and virulence information to predict the virulence of each isolate in the test set based on AGE presence or absence. Model performance on the test set (Table 2 and Fig. 4D) was comparable to the estimates made through nested cross-validation. For example, the test set accuracy of 0.72 was comparable to the mean nested cross-validation accuracy of 0.75 (95% CI, 0.69 to 0.80). This suggests that our predictive model of virulence is broadly applicable even when tested against geographically distinct isolates. Several of the misclassified isolates in the test set appear to be exceptions in virulence compared to their closest neighbor(s) in the core genome phylogenetic tree and to the accessory genome heatmap (Fig. 4A and C). Difficulty classifying these exceptional isolates is consistent with the notion that the model predictions are based on genomic signatures that perhaps approximate phylogenetic relationships. Closely related isolates that differ in virulence from the majority of their genomic neighbors would therefore be expected to be misclassified.

**TABLE 2:** Performance of random forest models trained using different genomic features against the 25 test isolates

Feature set	Accuracy	Sensitivity	Specificity	PPV^*a*^	AUC^*b*^	F1
AGEs	0.72	0.80	0.60	0.75	0.77	0.77
Core SNVs	0.72	0.67	0.80	0.83	0.69	0.74
8-mers	0.60	0.53	0.70	0.73	0.63	0.62
10-mers	0.68	0.73	0.60	0.73	0.72	0.73

a PPV, positive predictive value.

b AUC, area under the receiver operating characteristic curve.

While it was reassuring that the random forest model performed similarly against the test set as in nested cross-validation, we wanted to ensure that the accuracy observed did not simply occur by chance. We randomly permuted the predicted virulence of the 25 test set isolates to model the null distribution of test set accuracies that we would expect if no link between accessory genome content and virulence existed in the test set. After one million permutations, an accuracy of at least 0.72 was found in 53,476 cases (one-sided *P* = 0.053) (Fig. 4E). The test set performance observed is, therefore, unlikely if the accessory genome does not predict virulence. Limiting factors include the small sample size of the independent test set, as is evident from the discrete possible accuracies when the predictions were permuted, and that we would not expect the model to perform better against new data than it did during nested cross-validation.

### Addressing model limitations by removing isolates with intermediate levels of virulence.

**FIG 5:**
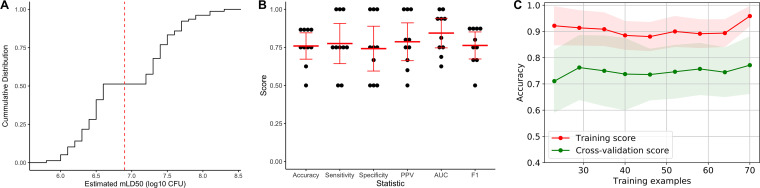
Performance of the random forest algorithm in predicting P. aeruginosa virulence from accessory genomic content when intermediate virulence isolates (middle third of estimated mLD_50_ values) were removed. (A) Cumulative distribution function of estimated mLD_50_ values after removing intermediate virulence isolates. Isolates with estimated mLD_50_ values less than the median value in the complete training set (red dashed line) were designated high virulence, with the remainder designated low virulence. (B) Nested 10-fold cross-validation performance of the random forest model, including accuracy, sensitivity, specificity, positive predictive value (PPV), area under the receiver operating characteristic curve (AUC), and F1 score. The results for each cross-validation fold are shown in black with the mean and 95% confidence interval of each statistic indicated in red. (C) Learning curve showing change in mean training accuracy (red line) and cross-validation accuracy (green line) with increasing training set sizes. Shading indicates the 95% confidence interval. Assessments at each number of training examples were through 10-fold nested cross-validation.

While the models generated thus far showed that the accessory genome is predictive of P. aeruginosa virulence in mice, limitations inherent to our binary classification of virulence may have constrained their performance. The first lies in the resolution of the mLD_50_ estimates used as the basis for these classes. Because of the practical limitations of testing over 100 isolates in mice, many isolates were tested with only two or three doses. This leads to uncertainty in the dose required to cause severe disease (Tables S2 and S3). Second, isolates with mLD_50_ estimates close to the cutoff may actually be quite similar, both in their virulence and in their genomic makeup, but still be assigned to different virulence classes. To assess the extent to which this ambiguity influenced the results, we repeated the machine learning pipeline using the random forest algorithm after removing intermediate-virulence isolates (the middle third of estimated mLD_50_ values). This enforced a greater separation of isolates classified as high and low virulence (Fig. 5A). Even with a third fewer training isolates, nested cross-validation performance was similar to when all training isolates were included, with a mean accuracy of 0.76 (95% CI, 0.67 to 0.85) (Fig. 5B). The learning curve, however, showed a greater distance between the training and cross-validation scores (Fig. 5C). This suggests a higher potential performance when intermediate virulence isolates are removed. The benefit of having a clearer boundary between high and low virulence would likely become apparent with a larger training set, though the number needed and the degree of improvement are unclear.

### Incorporating test set isolates into the accessory genome model.

After using the 25 additional isolates as an independent test set, we next examined their impact on nested cross-validation performance if they were included in the training set. As this changed the median estimated mLD_50_, we performed the modeling using both the median of the 115 training set isolates and the median of all 140 isolates as the cutoff for high/low virulence. The models performed similarly, both to each other and to the results seen with only the original training set. The mean nested cross-validation accuracy was 0.72 (95% CI, 0.65 to 0.79) when using the median mLD_50_ cutoff of the 115 training isolates and 0.69 (95% CI, 0.60 to 0.78) when using the median mLD_50_ cutoff of all 140 isolates (Fig. S4C and E). It is notable that adding an additional 25 isolates to the training set (and considering the new AGEs in these isolates) did not result in an improvement in model performance. The learning curves, however, showed greater overfitting of the model when the all-isolates median cutoff was used, with a larger separation between the training and cross-validation accuracies (Fig. S4D and F). This suggests the choice of cutoff between high- and low-virulence isolates may become more important with increasing training set sizes. Removing intermediate-virulence isolates resulted in similar nested cross-validation performance and learning curves to that seen when performing this analysis on the original training isolates (Fig. S4B, G, and H).

10.1128/mBio.01527-20.4 FIG S4 Performance of the random forest algorithm in predicting virulence from accessory genomic content when all 140 tested P. aeruginosa isolates were used to train the model. Cumulative distribution functions of estimated mLD_50_ values considering (A) all 140 tested isolates and (B) after removing intermediate virulence isolates. Isolates were designated high or low virulence based on whether their estimated mLD_50_ was lower than the median value in the training isolates (red dashed line) or all isolates (purple dashed line). Nested cross-validation performance when defining high virulence based on the median estimated mLD_50_ in the (C) training isolates, (E) all tested isolates, and (G) after removing intermediate virulence isolates, including accuracy, sensitivity, specificity, positive predictive value (PPV), area under the receiver operating characteristic curve (AUC), and F1 score. The results for each cross-validation fold are shown in black with the mean and 95% confidence interval of each statistic indicated in red. Learning curves showing change in mean training accuracy (red line) and cross-validation accuracy (green line) with increasing training set sizes when defining high virulence based on the median estimated mLD_50_ in the (D) training isolates, (F) all tested isolates, and (H) after removing intermediate virulence isolates. Shading indicates the 95% confidence interval. Assessments at each number of training examples were through 10-fold nested cross-validation. Download FIG S4, TIF file, 2.7 MB. Copyright © 2020 Pincus et al. 2020 Pincus et al. This content is distributed under the terms of the Creative Commons Attribution 4.0 International license.

### Modeling P. aeruginosa virulence with features incorporating core genome information.

Thus far we have shown that the accessory genome of P. aeruginosa is predictive of strain virulence. The accessory genome and core genome are correlated with each other, as can be seen from previous reports (4) and by comparing core and accessory genome measures of strain relatedness (Fig. 1B and C). As such, the accessory genome contains implicit information about the core genome. Still, it is possible that our focus on the accessory genome misses important core features predictive of virulence. To address this possibility, we defined our feature set in two additional ways and examined the performance of random forest models trained using these features. First, we considered core genome SNVs. Here, we used one-hot encoding in our machine learning pipeline to convert SNVs from nucleotides into binary variables interpretable by the algorithm. Second, we used whole-genome k-mer counts, which encode information about variability in both the accessory and core genome. k-mers are defined by dividing the genome into overlapping sequences of length *k*. We considered k-mer lengths of both 8 and 10 bp. Unlike the AGE feature set used previously, which considered the presence and absence of accessory elements, the k-mer feature sets additionally capture polymorphisms within these elements. We estimated the performance of approaches using these feature sets through nested cross-validation and then assessed how well final models built with each were able to predict the virulence of the 25 independent test set isolates.

**FIG 6:**
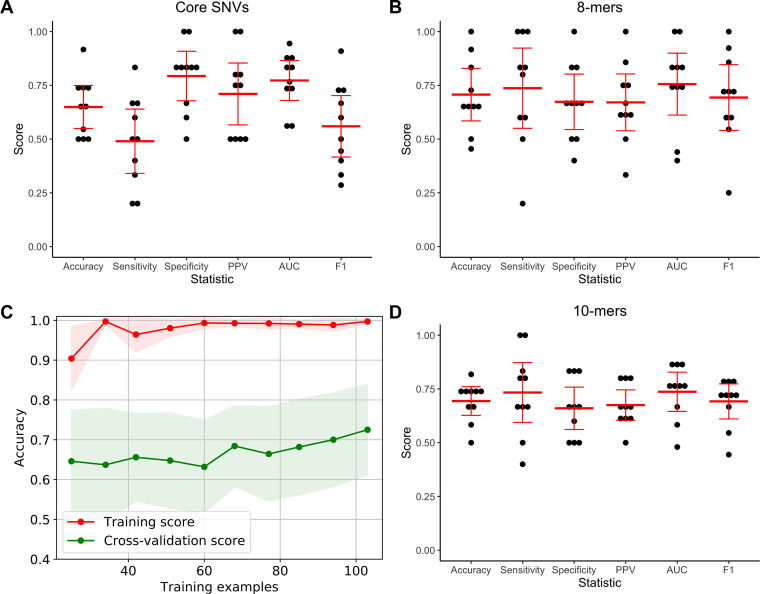
Performance of the random forest algorithm in predicting P. aeruginosa virulence when 8-mer counts, 10-mer counts, or core genome SNVs were used as model features. Nested cross-validation performance when using (A) core genome SNVs, (B) 8-mer counts, and (D) 10-mer counts, including accuracy, sensitivity, specificity, positive predictive value (PPV), area under the receiver operating characteristic curve (AUC), and F1 score. The results for each cross-validation fold are shown in black with the mean and 95% confidence interval of each statistic indicated in red. (C) Learning curve showing change in mean training accuracy (red line) and cross-validation accuracy (green line) when using 8-mer counts as features as increasing numbers of isolates are used to train the random forest model. Shading indicates the 95% confidence interval. Assessments at each number of training examples were through 10-fold nested cross-validation. Learning curves were not constructed when using core genome SNV or 10-mer counts as features for reasons of computational feasibility.

A random forest approach using core genome SNVs as features performed worse on average in nested cross-validation than when using accessory genomic features, with a mean accuracy of 0.65. However, its 95% confidence interval (0.55 to 0.75) still overlapped with those seen for the accessory genomic models (Fig. 6A). Therefore, some information important for determining virulence level may be missed by not considering the accessory genome. Another explanation is that more strains may be needed to model this substantially more complex feature set, as there were 440,116 core genome SNV loci detected in our training set. As the confidence intervals overlap, we must be careful drawing conclusions about the relative predictive power of the core and accessory genomes. The final model trained with core genome SNV features showed an accuracy of 0.72 on the independent test set. This was identical to the test set accuracy seen for the accessory genomic model but was associated with a lower sensitivity and higher specificity (Table 2). Despite the lower nested cross-validation accuracy of the core genome SNV model, we cannot say whether the accessory genome or core genome are superior in predicting virulence.

The random forest approach using k-mer counts as features performed similarly to the accessory genome models in nested cross-validation, with a nested cross-validation accuracy of 0.71 (95% CI, 0.58 to 0.83) when 8-mer counts were used and 0.69 (95% CI, 0.63 to 0.76) when 10-mer counts were used (Fig. 6B and D). This suggests that no additional predictive information was gained from incorporating core genome features, and that AGE presence/absence encodes the same information in a smaller feature set. The learning curve for the model trained on 8-mer counts showed overfitting, with a large discrepancy between the training and cross-validation accuracies (Fig. 6C). This suggests that performance would improve with a larger training set, and perhaps that the increased complexity of the 8-mer feature set makes it more difficult to learn from than the presence or absence of AGEs. The final model trained with 8-mer features showed an accuracy of 0.60 on the test set, while the final model trained on the 10-mer feature set showed an accuracy of 0.68 (Table 2). The performance of the 8-mer feature set was more variable in nested cross-validation, with a wider range in its 95% confidence interval, and it is possible that lower model stability contributed to its poorer performance against the test set.

## DISCUSSION

In this study, we have shown that a signal exists in the P. aeruginosa accessory genome that is predictive of an isolate’s virulence in a mouse model of infection. This finding was consistent across a variety of machine learning algorithms. Results for the random forest approach were validated using an independent test set of clinical isolates collected from a geographically distinct source, showing the broad applicability of the P. aeruginosa accessory genome in predicting virulence. We additionally showed that the core genome, alone or in combination with the accessory genome, is also predictive of virulence, but the ability of models trained on this information to generalize to the independent test set was less conclusive. These types of genetic features were substantially more complex, and models trained from them may benefit from increasing sample size. The machine learning analyses conducted here serve as a framework to further investigate the relationship between the genome of a bacterium and its phenotype.

The random forest model trained on accessory genomic information classified isolates as high- or low-virulence based on a diffuse genomic signature rather than by detecting a small number of virulence or antivirulence factors. The genomic signature detected may approximate lineage, echoing the recent finding that genomic neighbors are highly predictive of antimicrobial resistance in Streptococcus pneumoniae and Neisseria gonorrhoeae (27). Supporting this conclusion is the finding that individual AGEs showed low importance in random forest model predictions and that models could be built using only a random tenth of the total AGEs without a dramatic loss of performance. Furthermore, some of the misclassified test set strains were virulence outliers relative to their phylogenetic neighbors. Still, information encoded in the genome is not necessarily simply phylogenetic. This was shown in a recent study by Khaledi et al. (26) using genomic and transcriptomic features to predict antimicrobial resistance in P. aeruginosa. They tested the influence of phylogenetics on their resistance predictions through “block cross-validation,” in which they enforced the requirement that training and cross-validation folds contained nonoverlapping sequence types. This resulted in modest reductions in performance but showed that resistance could be predicted even when testing against phylogenetically distinct isolates (26). Future studies should determine the extent to which P. aeruginosa virulence is linked to or independent of phylogenetics.

While individual AGEs showed low importance in model predictions, it is relevant that all of the 10 most important AGEs included in our model were associated with low virulence. This supports the earlier finding that the presence of specific P. aeruginosa accessory genes can reduce virulence in C. elegans and that active CRISPR systems, which would limit acquisition of foreign DNA and new AGEs, are associated with higher virulence in that model (16). While certain AGEs enhance virulence (15), many AGEs (e.g., parasitic phages, plasmids, or ICEs) may decrease virulence through mechanisms such as dysregulation of regulatory networks, insertion into important genes, or imposition of an additional metabolic burden. The latter possibility could be assessed by examining the *in vitro* growth rate of the isolates included in this study and determining whether AGEs predictive of low virulence were associated with slower growth. In addition, it could be determined whether deletion of these AGEs resulted in an increased growth rate. This should be accompanied by a systematic investigation into the types of AGEs that are associated with low and high virulence. We focused on virulence in a mouse model of acute infection, and therefore certain bacterial genetic factors important in the hospital setting may not apply. Antimicrobial resistance, for example, can be an important prognostic factor for patient outcomes (37, 38) but would not be relevant in this model. Future studies should examine the types of AGEs that are associated with, and ultimately causal of, both increased and decreased virulence, and how this differs between infection models.

Our random forest model built on accessory genomic features showed similar performance in nested cross-validation as when the model was applied to an independent test set of 25 isolates. By looking at the test set isolates that were classified incorrectly, we can learn why the model sometimes failed. Some incorrect predictions may be because of mLD_50_ values near the threshold between high and low virulence, leading to ambiguity in their true virulence level. An example of this scenario is the isolate PASP518, whose estimated mLD_50_ of 7.0 log_10_ CFU is near the cutoff of less than 6.9 log_10_ CFU for high virulence. This highlights inherent limitations of this study, namely, that virulence exists on a continuum not neatly divided into binary classes and that the limited number of mice tested for each isolate creates uncertainty in the estimations of the mLD_50_ values. Both of these factors could decrease the accuracy of our models. To address these limitations, we examined how the model performs when excluding intermediate virulence isolates. Under this condition, a random forest approach performed similarly in nested cross-validation with a third fewer samples; learning curve analysis showed a potential for higher accuracy with increasing sample size (Fig. 5). On the other hand, as mentioned above, some of the incorrect predictions in the test set were exceptions in virulence compared to closely related isolates. An increased sample size may ameliorate the problem of isolates being misclassified by allowing for finer resolution of subgroups that are associated with high or low virulence, especially if the model were able to learn new and more discriminatory patterns of features. Learning curve analysis for the random forest approach (Fig. 3A) suggests that the impact of adding more isolates would be limited, but this cannot account for new or more predictive features that could arise from increasing the amount of genetic data available.

As whole-genome sequencing becomes an increasingly routine component of clinical microbiology practice, it will create the opportunity to risk stratify patients based on the genome of an infecting bacterium and influence treatment decisions in real-time. The ability of the genome to predict antibiotic resistance has been established (21, 22, 24, 26, 27), opening the door for sequencing to supplement or replace traditional antimicrobial susceptibility testing. This study serves as a proof of concept that the P. aeruginosa genome can be used to predict its pathogenicity. Future studies are needed to expand beyond virulence in mice and to provide a more complete understanding of the role genetic variation plays the ability of P. aeruginosa to cause disease. An area of particular interest is in predicting patient outcomes from the genome of an infecting isolate. Large retrospective studies using archived isolates with corresponding clinical data would allow for exploration of the relative importance that bacterial and patient factors play in predicting patient outcomes, as has been shown for specific S. aureus clones (29). This could improve the sophistication of current diagnostics and allow clinicians to rapidly identify patients at highest risk for poor outcomes.

## MATERIALS AND METHODS

### Bacterial isolates.

A training set of P. aeruginosa isolates for use in the machine learning analyses was established as follows. A total of 98 isolates previously collected at NMH in Chicago, IL, USA, from 1999 to 2003 from adults with P. aeruginosa bacteremia (32) were selected after the exclusion of 2 isolates that had been collected from patients with a history of cystic fibrosis. An additional 17 isolates from pediatric patients with Shanghai fever collected at Chang Gung Children’s Hospital in Taiwan from 2003 to 2008 (33) were included. This yielded a training set size of 115 isolates. A genetically diverse independent test set of 25 isolates was selected from a larger cohort of isolates collected from patients with bacteremia in Spain between 2008 and 2009 (36) (see Table S1 in the supplemental material).

### Mouse model of bacteremia.

Female 6- to 9-week-old BALB/c mice were infected via tail vein injection in a model of bacteremia as previously described (33). Isolates were plated from freezer stocks onto lysogeny broth (LB) agar, and single colonies were inoculated into MINS broth (39) and grown overnight at 37°C. Overnight cultures were then subcultured in fresh MINS broth for approximately 3 h at 37°C. Cultures were resuspended in phosphate-buffered saline (PBS) before dilution to the target dose, and 50 μl was injected into each mouse via the tail vein. Inocula, in CFU, were then determined by serial dilution, plating, and colony counts. Mice were monitored for the development of severe disease over 5 days, with mice exhibiting endpoint disease euthanized and scored as dead. Each isolate was tested at a minimum of 2 doses, with 3 to 5 mice per dose (minimum 9 total mice per isolate) (Table S2). Many of the mouse experiments included in this study were previously reported as part of other studies. In particular, the majority of experiments with the NMH strains were performed as part of Allen et al. (15). Some experiments with the Taiwan isolates PAC1 and PAC6 were performed as part of Chuang et al. (33).

A modified 50% lethal dose (mLD_50_) for each isolate was estimated from the above-described experiments using the *drc* package (v3.0-1) (40) in R (v3.6.1) (41). One outlier experiment for strain S2, which caused 20% mortality at a dose of ∼7.2 log_10_ CFU, was excluded because doses of ∼6.3 and ∼6.8 log_10_ CFU caused 80% and 100% mortality, respectively, in other experiments. Percent mortality as a function of dose (in units of log_10_ CFU) was modeled using a two-parameter log-logistic function and binomial data type. These models were used to estimate the mLD_50_ for each isolate, which was then rounded to the nearest tenth (see Table S3 in the supplemental material). Isolates with rounded mLD_50_ estimates below the median were classified as high virulence, with the remainder classified as low virulence.

All experiments were approved by the Northwestern University Institutional Animal Care and Use Committee in compliance with all relevant ethical regulations for animal testing and research.

### Whole-genome sequencing and assembly.

Short-read whole-genome sequencing was performed for all isolates using either the Illumina HiSeq or MiSeq platform to generate paired-end reads. Reads were trimmed using Trimmomatic (v0.36) (42) with Nextera adapter removal, a sliding window size of 4 bp with an average quality threshold of 15, and a minimum trimmed read length of 36 bp. Draft genomes were assembled from trimmed paired-end reads using SPAdes (v3.9.1) (43) with the “careful” and “automatic read coverage cutoff” options. Draft genomes were further filtered to remove contigs shorter than 200 bp, with less than 5-fold mean read coverage, or with alignment to phiX. Even using only trimmed reads, the mean coverage of each filtered assembly was at least 24-fold. Many of the whole-genome sequences used in this study were previously reported as parts of other studies (15, 44, 45). Draft genomes originally assembled through different methodologies were reassembled as described above.

For several genomes (PABL012, PABL017, PABL048, PAC1, and PAC6), long-read sequencing and hybrid assembly were performed. Briefly, genomes were sequenced on the PacBio RS II platform. Raw data were assembled using the HGAP assembler (SMRT Analysis v2.3.0), Canu assembler (v1.2) (46), and Celera assembler (v8.2) (47), all using default settings. Contigs were combined and circularized using Circlator (v1.5.1) (48). Assemblies were polished using Quiver (SMRT Analysis v2.3.0). Indel errors were corrected using Pilon (v1.21) (49) using paired-end reads generated on the Illumina HiSeq or MiSeq platforms. The complete genome for PABL048 was generated as part of a previous study (44).

### Phylogenetic analysis.

kSNP (v3.0.21) was used to generate 95% core genome parsimony phylogenetic trees for both 115 isolates in the training set and all 140 isolates in the training and test sets, using fasta files as input. The Kchooser program was used to select the optimum k-mer size of 21, and single-nucleotide polymorphism (SNP) loci present in at least 95% of input genomes were used to make the trees (50). The phylogenetic trees were annotated and plots generated using iTOL (v4) (51).

### Accessory genome determination.

Accessory genomes for the 115 P. aeruginosa isolates in the training set were determined using the programs Spine (v0.3.2), AGEnt (v0.3.1), and ClustAGE (v0.8) (7, 52). Spine was used with Prokka-annotated (53) GenBank files for each isolate as the input to generate a core genome of sequences present in at least 95% of isolates. AGEnt was then used to determine the accessory genome of each isolate based on comparison to the core genome. The accessory genomes of all 115 isolates were then compared using ClustAGE to identify shared sequences using an 85% identity cutoff. ClustAGE identifies the longest continuous accessory sequences as “bins” and the portions of these bins that differ from isolate to isolate as “subelements” (15, 52). As part of this process, the read correction feature of ClustAGE was used to identify sequences present in the original sequencing reads that were missed during genome assembly. All perfectly correlated subelements identified through clustAGE were collapsed into a single feature, termed a “unique group (of subelements).” For the purpose of this study, accessory genomic elements (AGEs) were defined as all unique groups totaling ≥200 bp. A data frame of all AGEs in the training isolates served as the accessory genome feature set in subsequent machine learning analyses. To generate AGE features present in all genomes (both the original training and test sets), this process was repeated using all 140 P. aeruginosa isolates as the input.

To determine which AGEs from the training set were present in the test set, clustAGE was run using the training set read-corrected subelement sequences (for all subelements of ≥50 bp) from the 115 training isolates as a reference AGE set with the “–AGE” option and compared to the draft genomes of all isolates in the test set, with read correction to identify any sequences present that were not included in draft genome assembly. This identified which portions of each subelement were found in the test set with an 85% identity cutoff. An AGE (defined as a unique group of subelements) was called as present if at least 85% of the screened length was detected.

To examine the relationships between accessory genomes in the training isolates, their AGE content was compared using the subelement_to_tree.pl utility from ClustAGE. This calculated the Bray-Curtis dissimilarity between each isolate based on AGE presence or absence, with the impact of each AGE weighted by its length. A neighbor-joining tree was constructed from 1,000 bootstrap replicates using the matrix of Bray-Curtis dissimilarities. For consistency with the definition of AGE used in this study, unique groups of subelements were used as input. The neighbor-joining tree and associated heatmap of Bray-Curtis dissimilarities were annotated and visualized with iTOL (v4) (51). To examine the accessory genomic relatedness of the 25 test set isolates based on training-set derived AGEs, the training set AGE calls defined above were added, and Bray-Curtis dissimilarity calculations and neighbor joining tree construction were repeated. To further evaluate the relationships between accessory genomes, multiple correspondence analysis (MCA) was performed based on the presence or absence of AGEs in the 115 training isolates. Additionally, MCA was perfumed considering which of the training isolate AGEs were identified in all 140 isolates. MCA was performed in R (v3.6.1) (41) using the *FactoMineR* (v2.3) (54) package and visualized using the *factoextra* (v1.0.6) package.

### Sequence alignment and core SNV calling.

Sequence alignment of paired-end Illumina reads for each genome to the reference genome PAO1 (RefSeq accession number NC_002516) was performed as previously described (44). Briefly, reads were trimmed with Trimmomatic (v0.36) (42) and aligned to PAO1 with BWA (v0.7.15) (55). Loci passing inclusion criteria were called as having the PAO1 base or a SNV base for each genomic position, with the remainder of positions converted to gaps. PAO1 alignments for all 115 training isolates were concatenated, SNV positions present in fewer than 95% of genomes were filtered, and invariant sites were then removed. This core variant SNV alignment was used as the SNV feature set in subsequent machine learning analyses, with a one-hot encoding step added to the pipeline to convert SNV loci into multiple binary variables. This feature set was defined in the test set by considering the genomic positions identified as variant in the training set. By extracting the sequence present at these variant positions in the PAO1 alignments for each of the 25 test set isolates, we created a SNV feature set corresponding to that used in the training set.

### k-mer counts.

k-mer counts (using either 8- or 10-bp k-mers) were determined for each genome using KMC3 (v3.0.0) (56). All k-mers occurring at least once in each genome’s fasta file were identified using the kmc application, and a count file was generated using the kmc_dump application. All unique k-mers identified in the training set of 115 P. aeruginosa genomes were used to construct a data frame of k-mer counts for each genome. This served as the k-mer feature set in subsequent machine learning analyses. This feature set was defined in the 25 test set isolates by considering the counts of all k-mers previously identified in the training set.

### Predicting virulence based on genomic features.

Machine learning analyses were performed using the sci-kit learn library (v0.21.2) (57) in Python (v3.6.9). The general workflow for the machine learning pipeline is described in Fig. S2 in the supplemental material. A training data set of features (AGEs, k-mers, or core SNVs) and labels (high/low virulence) was defined. A machine learning algorithm (random forest, l2-regularized logistic regression, elastic net logistic regression, or support vector classifier) was chosen, and a grid of relevant hyperparameters to test were defined. A machine learning model was then trained using the selected algorithm, with hyperparameter tuning performed through grid search cross-validation. A 10-fold stratified cross-validation strategy was used. This generated a final model that was used to predict the virulence class of new isolates. Concurrently the generalization performance of this model was estimated through nested cross-validation. In this process, grid search cross-validation was performed within an outer 10-fold stratified cross-validation loop. The performance of a grid search cross-validation tuned model against each cross-validation fold was determined (including accuracy, sensitivity, specificity, positive predictive value [PPV], area under the receiver operating characteristic curve [AUC], and F1 score). The mean and 95% confidence interval of the nested cross-validation results were determined and plotted with the values for each fold using R (v3.6.1) (41) with the *tidyverse* library suite (v1.2.1) (58).

For the random forest algorithm, the number of trees was set to 10,000 and “max_features,” “min_samples_split,” “min_samples_leaf,” “criterion,” and “max_depth” were varied as hyperparameters during grid search cross-validation. The logistic regression algorithm was considered using l2 regularization (penalty = “l2”) and elastic net regularization (penalty = “elasticnet”) separately. For l2-regularized logistic regression, the “lbfgs” solver was used, “max_iter” was set to 10,000, and “C” was varied as a hyperparameter during grid-search cross-validation. For elastic net logistic regression, the “saga” solver was used, “max_iter” was set to 10,000, and “C” and “l1_ratio” were varied as hyperparameters. For the support vector classifier algorithm, the radial basis function kernel was used, and “C” and “gamma” were varied as hyperparameters during grid search cross-validation.

In some cases, learning curves were created to examine how training and nested cross-validation accuracy varied with increasing training test size. For this, the data set was split into training and cross-validation folds through 10-fold stratified cross-validation. Subsets of examples ranging from 25 to 100% of the training fold size were then drawn from each training fold. On each subset, a model was trained through the grid search cross-validation approach described above. The mean and 95% confidence interval for training and cross-validation accuracies at each number of examples were then determined and plotted.

### Random forest permutation importance.

Out-of-bag permutation importance for the random forest model of virulence based on accessory genomic content trained on the complete training set of 115 P. aeruginosa isolates was determined using the *rfpimp* (v1.3.4) Python package (https://github.com/parrt/random-forest-importances). This measures the decrease in accuracy in predicting out-of-bag samples (samples not used to train a given decision tree in the random forest) if a feature is randomly permuted. As the impact of permuting a given feature on model accuracy may depend on how it is permuted, this process was repeated a total of 100 times to determine a mean permutation importance (see Table S4 in the supplemental material). The putative annotation of the top 10 AGEs identified by permutation importance was determined by blast search of subelement sequences against the *Pseudomonas* Genome Database (59) and inclusion of the annotation of any open reading frame (ORF) for which at least 50 bp were contained in the AGE.

### Evaluating random forest model performance with an independent test set.

The random forest model trained on AGE presence/absence in the 115 training isolates was tested against the independent test set of 25 isolates. The training set AGEs identified in these 25 isolates were used as features, and the predicted virulence classes were compared to the actual virulence for these isolates. This was used to estimate testing accuracy, sensitivity, specificity, positive predictive value, area under the receiver operating characteristic curve, and F1 score and to plot the receiver operating characteristic curve. This approach was also used to assess the performance of random forest models trained on core genome SNVs, 8-mers, and 10-mers against the independent test set of 25 isolates.

For the accessory genome model, the probability of seeing the observed test set accuracy by chance if there was no true association between the predicted virulence (and therefore accessory genome) of an isolate and its true virulence was estimated through permutation testing. The predicted virulence classes for the 25 test isolates were randomly permuted 1 million times and used to create a null distribution of possible model accuracies. The observed test set accuracy was compared to this null distribution to estimate a one-sided *P* value.

### Data availability.

BioSample accessions numbers for all isolates used in this study are listed in Table S1 in the supplemental material. For all isolates, the version of the genome assemblies used in this study are available on GitHub. Input data for machine learning analyses (including all AGE, core SNV, and k-mer feature sets) are also available on GitHub (https://github.com/nathanpincus/PA_Virulence_Prediction). Code used for machine learning analyses in this study, including details on hyperparameters used during grid search cross-validation and for plotting the results are available on GitHub (https://github.com/nathanpincus/PA_Virulence_Prediction).

## References

[1] Talbot GH, Bradley J, Edwards JE, Gilbert D, Scheld M, Bartlett JG, Antimicrobial Availability Task Force of the Infectious Diseases Society of America. 2006. Bad bugs need drugs: an update on the development pipeline from the Antimicrobial Availability Task Force of the Infectious Diseases Society of America. Clin Infect Dis 42:657–668. doi:10.1086/499819. 16447111

[2] Gellatly SL, Hancock REW. 2013. *Pseudomonas aeruginosa*: new insights into pathogenesis and host defenses. Pathog Dis 67:159–173. doi:10.1111/2049-632X.12033. 23620179

[3] Silby MW, Winstanley C, Godfrey SAC, Levy SB, Jackson RW. 2011. *Pseudomonas* genomes: diverse and adaptable. FEMS Microbiol Rev 35:652–680. doi:10.1111/j.1574-6976.2011.00269.x. 21361996

[4] Ozer EA, Nnah E, Didelot X, Whitaker RJ, Hauser AR. 2019. The population structure of *Pseudomonas aeruginosa* is characterized by genetic isolation of *exoU*^+^ and *exoS*^+^ lineages. Genome Biol Evol 11:1780–1796. doi:10.1093/gbe/evz119. 31173069 PMC6690169

[5] Freschi L, Vincent AT, Jeukens J, Emond-Rheault J-G, Kukavica-Ibrulj I, Dupont M-J, Charette SJ, Boyle B, Levesque RC. 2019. The *Pseudomonas aeruginosa* pan-genome provides new insights on its population structure, horizontal gene transfer, and pathogenicity. Genome Biol Evol 11:109–120. doi:10.1093/gbe/evy259. 30496396 PMC6328365

[6] Kung VL, Ozer EA, Hauser AR. 2010. The accessory genome of *Pseudomonas aeruginosa*. Microbiol Mol Biol Rev 74:621–641. doi:10.1128/MMBR.00027-10. 21119020 PMC3008168

[7] Ozer EA, Allen JP, Hauser AR. 2014. Characterization of the core and accessory genomes of *Pseudomonas aeruginosa* using bioinformatic tools Spine and AGEnt. BMC Genomics 15:737. doi:10.1186/1471-2164-15-737. 25168460 PMC4155085

[8] Mosquera-Rendón J, Rada-Bravo AM, Cárdenas-Brito S, Corredor M, Restrepo-Pineda E, Benítez-Páez A. 2016. Pangenome-wide and molecular evolution analyses of the *Pseudomonas aeruginosa* species. BMC Genomics 17:45. doi:10.1186/s12864-016-2364-4. 26754847 PMC4710005

[9] LaFayette SL, Houle D, Beaudoin T, Wojewodka G, Radzioch D, Hoffman LR, Burns JL, Dandekar AA, Smalley NE, Chandler JR, Zlosnik JE, Speert DP, Bernier J, Matouk E, Brochiero E, Rousseau S, Nguyen D. 2015. Cystic fibrosis-adapted *Pseudomonas aeruginosa* quorum sensing *lasR* mutants cause hyperinflammatory responses. Sci Adv 1:e1500199. doi:10.1126/sciadv.1500199. 26457326 PMC4597794

[10] Lorè NI, Cigana C, De Fino I, Riva C, Juhas M, Schwager S, Eberl L, Bragonzi A. 2012. Cystic fibrosis-niche adaptation of *Pseudomonas aeruginosa* reduces virulence in multiple infection hosts. PLoS One 7:e35648. doi:10.1371/journal.pone.0035648. 22558188 PMC3338451

[11] Ho Sui SJ, Fedynak A, Hsiao WWL, Langille MGI, Brinkman FSL. 2009. The association of virulence factors with genomic islands. PLoS One 4:e8094. doi:10.1371/journal.pone.0008094. 19956607 PMC2779486

[12] He J, Baldini RL, Déziel E, Saucier M, Zhang Q, Liberati NT, Lee D, Urbach J, Goodman HM, Rahme LG. 2004. The broad host range pathogen *Pseudomonas aeruginosa* strain PA14 carries two pathogenicity islands harboring plant and animal virulence genes. Proc Natl Acad Sci U S A 101:2530–2535. doi:10.1073/pnas.0304622101. 14983043 PMC356984

[13] Battle SE, Meyer F, Rello J, Kung VL, Hauser AR. 2008. Hybrid pathogenicity island PAGI-5 contributes to the highly virulent phenotype of a *Pseudomonas aeruginosa* isolate in mammals. J Bacteriol 190:7130–7140. doi:10.1128/JB.00785-08. 18757543 PMC2580712

[14] Harrison EM, Carter MEK, Luck S, Ou H-Y, He X, Deng Z, O'Callaghan C, Kadioglu A, Rajakumar K. 2010. Pathogenicity islands PAPI-1 and PAPI-2 contribute individually and synergistically to the virulence of *Pseudomonas aeruginosa* strain PA14. Infect Immun 78:1437–1446. doi:10.1128/IAI.00621-09. 20123716 PMC2849418

[15] Allen JP, Ozer EA, Minasov G, Shuvalova L, Kiryukhina O, Satchell KJF, Hauser AR. 2020. A comparative genomics approach identifies contact-dependent growth inhibition as a virulence determinant. Proc Natl Acad Sci U S A 117:6811–6821. doi:10.1073/pnas.1919198117. 32156726 PMC7104216

[16] Vasquez-Rifo A, Veksler-Lublinsky I, Cheng Z, Ausubel FM, Ambros V. 2019. The *Pseudomonas aeruginosa* accessory genome elements influence virulence towards *Caenorhabditis elegans*. Genome Biol 20:270. doi:10.1186/s13059-019-1890-1. 31823826 PMC6902481

[17] Brockhurst MA, Harrison E, Hall JPJ, Richards T, McNally A, MacLean C. 2019. The ecology and evolution of pangenomes. Curr Biol 29:R1094–R1103. doi:10.1016/j.cub.2019.08.012. 31639358

[18] Hauser AR, Kang PJ, Engel JN. 1998. PepA, a secreted protein of *Pseudomonas aeruginosa*, is necessary for cytotoxicity and virulence. Mol Microbiol 27:807–818. doi:10.1046/j.1365-2958.1998.00727.x. 9515706

[19] Shaver CM, Hauser AR. 2004. Relative contributions of *Pseudomonas aeruginosa* ExoU, ExoS, and ExoT to virulence in the lung. Infect Immun 72:6969–6977. doi:10.1128/IAI.72.12.6969-6977.2004. 15557619 PMC529154

[20] Libbrecht MW, Noble WS. 2015. Machine learning applications in genetics and genomics. Nat Rev Genet 16:321–332. doi:10.1038/nrg3920. 25948244 PMC5204302

[21] Davis JJ, Boisvert S, Brettin T, Kenyon RW, Mao C, Olson R, Overbeek R, Santerre J, Shukla M, Wattam AR, Will R, Xia F, Stevens R. 2016. Antimicrobial resistance prediction in PATRIC and RAST. Sci Rep 6:27930. doi:10.1038/srep27930. 27297683 PMC4906388

[22] Nguyen M, Long SW, McDermott PF, Olsen RJ, Olson R, Stevens RL, Tyson GH, Zhao S, Davis JJ. 2018. Using machine learning to predict antimicrobial MICs and associated genomic features for nontyphoidal *Salmonella*. J Clin Microbiol 57:e01260-18. doi:10.1128/JCM.01260-18. PMC6355527 30333126

[23] Kavvas ES, Catoiu E, Mih N, Yurkovich JT, Seif Y, Dillon N, Heckmann D, Anand A, Yang L, Nizet V, Monk JM, Palsson BO. 2018. Machine learning and structural analysis of *Mycobacterium tuberculosis* pan-genome identifies genetic signatures of antibiotic resistance. Nat Commun 9:4306. doi:10.1038/s41467-018-06634-y. 30333483 PMC6193043

[24] Chen ML, Doddi A, Royer J, Freschi L, Schito M, Ezewudo M, Kohane IS, Beam A, Farhat M. 2019. Beyond multidrug resistance: leveraging rare variants with machine and statistical learning models in *Mycobacterium tuberculosis* resistance prediction. EBioMedicine 43:356–369. doi:10.1016/j.ebiom.2019.04.016. 31047860 PMC6557804

[25] Hyun JC, Kavvas ES, Monk JM, Palsson BO. 2020. Machine learning with random subspace ensembles identifies antimicrobial resistance determinants from pan-genomes of three pathogens. PLoS Comput Biol 16:e1007608. doi:10.1371/journal.pcbi.1007608. 32119670 PMC7067475

[26] Khaledi A, Weimann A, Schniederjans M, Asgari E, Kuo T-H, Oliver A, Cabot G, Kola A, Gastmeier P, Hogardt M, Jonas D, Mofrad MRK, Bremges A, McHardy AC, Häussler S. 2020. Predicting antimicrobial resistance in *Pseudomonas aeruginosa* with machine learning-enabled molecular diagnostics. EMBO Mol Med 12:e10264. doi:10.15252/emmm.201910264. 32048461 PMC7059009

[27] Břinda K, Callendrello A, Ma KC, MacFadden DR, Charalampous T, Lee RS, Cowley L, Wadsworth CB, Grad YH, Kucherov G, O’Grady J, Baym M, Hanage WP. 2020. Rapid inference of antibiotic resistance and susceptibility by genomic neighbour typing. Nat Microbiol 5:455–464. doi:10.1038/s41564-019-0656-6. 32042129 PMC7044115

[28] Laabei M, Recker M, Rudkin JK, Aldeljawi M, Gulay Z, Sloan TJ, Williams P, Endres JL, Bayles KW, Fey PD, Yajjala VK, Widhelm T, Hawkins E, Lewis K, Parfett S, Scowen L, Peacock SJ, Holden M, Wilson D, Read TD, van den Elsen J, Priest NK, Feil EJ, Hurst LD, Josefsson E, Massey RC. 2014. Predicting the virulence of MRSA from its genome sequence. Genome Res 24:839–849. doi:10.1101/gr.165415.113. 24717264 PMC4009613

[29] Recker M, Laabei M, Toleman MS, Reuter S, Saunderson RB, Blane B, Török ME, Ouadi K, Stevens E, Yokoyama M, Steventon J, Thompson L, Milne G, Bayliss S, Bacon L, Peacock SJ, Massey RC. 2017. Clonal differences in *Staphylococcus aureus* bacteraemia-associated mortality. Nat Microbiol 2:1381–1388. doi:10.1038/s41564-017-0001-x. 28785103

[30] Wheeler NE, Gardner PP, Barquist L. 2018. Machine learning identifies signatures of host adaptation in the bacterial pathogen *Salmonella enterica*. PLoS Genet 14:e1007333. doi:10.1371/journal.pgen.1007333. 29738521 PMC5940178

[31] Cornforth DM, Dees JL, Ibberson CB, Huse HK, Mathiesen IH, Kirketerp-Møller K, Wolcott RD, Rumbaugh KP, Bjarnsholt T, Whiteley M. 2018. *Pseudomonas aeruginosa* transcriptome during human infection. Proc Natl Acad Sci U S A 115:E5125–E5134. doi:10.1073/pnas.1717525115. 29760087 PMC5984494

[32] Scheetz MH, Hoffman M, Bolon MK, Schulert G, Estrellado W, Baraboutis IG, Sriram P, Dinh M, Owens LK, Hauser AR. 2009. Morbidity associated with *Pseudomonas aeruginosa* bloodstream infections. Diagn Microbiol Infect Dis 64:311–319. doi:10.1016/j.diagmicrobio.2009.02.006. 19345039 PMC2693471

[33] Chuang C-H, Wang Y-H, Chang H-J, Chen H-L, Huang Y-C, Lin T-Y, Ozer EA, Allen JP, Hauser AR, Chiu C-H. 2014. Shanghai fever: a distinct *Pseudomonas aeruginosa* enteric disease. Gut 63:736–743. doi:10.1136/gutjnl-2013-304786. 23943780 PMC3995289

[34] Mathee K, Narasimhan G, Valdes C, Qiu X, Matewish JM, Koehrsen M, Rokas A, Yandava CN, Engels R, Zeng E, Olavarietta R, Doud M, Smith RS, Montgomery P, White JR, Godfrey PA, Kodira C, Birren B, Galagan JE, Lory S. 2008. Dynamics of *Pseudomonas aeruginosa* genome evolution. Proc Natl Acad Sci U S A 105:3100–3105. doi:10.1073/pnas.0711982105. 18287045 PMC2268591

[35] Nguyen M, Olson R, Shukla M, VanOeffelen M, Davis JJ. 2020. Predicting antimicrobial resistance using conserved genes. bioRxiv doi:10.1101/2020.04.29.068254 PMC7595632 33075053

[36] Peña C, Suarez C, Gozalo M, Murillas J, Almirante B, Pomar V, Aguilar M, Granados A, Calbo E, Rodríguez-Baño J, Rodríguez F, Tubau F, Martínez-Martínez L, Oliver A, Spanish Network for Research in Infectious Diseases (REIPI). 2012. Prospective multicenter study of the impact of carbapenem resistance on mortality in *Pseudomonas aeruginosa* bloodstream infections. Antimicrob Agents Chemother 56:1265–1272. doi:10.1128/AAC.05991-11. 22155832 PMC3294876

[37] Peña C, Cabot G, Gómez-Zorrilla S, Zamorano L, Ocampo-Sosa A, Murillas J, Almirante B, Pomar V, Aguilar M, Granados A, Calbo E, Rodríguez-Baño J, Rodríguez-López F, Tubau F, Martínez-Martínez L, Oliver A, Spanish Network for Research in Infectious Diseases (REIPI). 2015. Influence of virulence genotype and resistance profile in the mortality of *Pseudomonas aeruginosa* bloodstream infections. Clin Infect Dis 60:539–548. doi:10.1093/cid/ciu866. 25378459

[38] Morata L, Cobos-Trigueros N, Martínez JA, Soriano Á, Almela M, Marco F, Sterzik H, Núñez R, Hernández C, Mensa J. 2012. Influence of multidrug resistance and appropriate empirical therapy on the 30-day mortality rate of *Pseudomonas aeruginosa* bacteremia. Antimicrob Agents Chemother 56:4833–4837. doi:10.1128/AAC.00750-12. 22751533 PMC3421866

[39] Nicas TI, Iglewski BH. 1984. Isolation and characterization of transposon-induced mutants of *Pseudomonas aeruginosa* deficient in production of exoenzyme S. Infect Immun 45:470–474. doi:10.1128/IAI.45.2.470-474.1984. 6086529 PMC263264

[40] Ritz C, Baty F, Streibig JC, Gerhard D. 2015. Dose-response analysis using R. PLoS One 10:e0146021. doi:10.1371/journal.pone.0146021. 26717316 PMC4696819

[41] R Core Team. 2019. R: a language and environment for statistical computing. R Foundation for Statistical Computing, Vienna, Austria. https://www.R-project.org/.

[42] Bolger AM, Lohse M, Usadel B. 2014. Trimmomatic: a flexible trimmer for Illumina sequence data. Bioinformatics 30:2114–2120. doi:10.1093/bioinformatics/btu170. 24695404 PMC4103590

[43] Bankevich A, Nurk S, Antipov D, Gurevich AA, Dvorkin M, Kulikov AS, Lesin VM, Nikolenko SI, Pham S, Prjibelski AD, Pyshkin AV, Sirotkin AV, Vyahhi N, Tesler G, Alekseyev MA, Pevzner PA. 2012. SPAdes: a new genome assembly algorithm and its applications to single-cell sequencing. J Comput Biol 19:455–477. doi:10.1089/cmb.2012.0021. 22506599 PMC3342519

[44] Pincus NB, Bachta KER, Ozer EA, Allen JP, Pura ON, Qi C, Rhodes NJ, Marty FM, Pandit A, Mekalanos JJ, Oliver A, Hauser AR. 2019. Long-term persistence of an extensively drug resistant subclade of globally distributed *Pseudomonas aeruginosa* clonal complex 446 in an academic medical center. Clin Infect Dis doi:10.1093/cid/ciz973. PMC7486844 31583403

[45] Bachta KER, Allen JP, Cheung BH, Chiu C-H, Hauser AR. 2020. Systemic infection facilitates transmission of *Pseudomonas aeruginosa* in mice. Nat Commun 11:543. doi:10.1038/s41467-020-14363-4. 31992714 PMC6987207

[46] Koren S, Walenz BP, Berlin K, Miller JR, Bergman NH, Phillippy AM. 2017. Canu: scalable and accurate long-read assembly via adaptive k-mer weighting and repeat separation. Genome Res 27:722–736. doi:10.1101/gr.215087.116. 28298431 PMC5411767

[47] Myers EW, Sutton GG, Delcher AL, Dew IM, Fasulo DP, Flanigan MJ, Kravitz SA, Mobarry CM, Reinert KHJ, Remington KA, Anson EL, Bolanos RA, Chou H-H, Jordan CM, Halpern AL, Lonardi S, Beasley EM, Brandon RC, Chen L, Dunn PJ, Lai Z, Liang Y, Nusskern DR, Zhan M, Zhang Q, Zheng X, Rubin GM, Adams MD, Venter JC. 2000. A whole-genome assembly of *Drosophila*. Science 287:2196–2204. doi:10.1126/science.287.5461.2196. 10731133

[48] Hunt M, Silva ND, Otto TD, Parkhill J, Keane JA, Harris SR. 2015. Circlator: automated circularization of genome assemblies using long sequencing reads. Genome Biol 16:294. doi:10.1186/s13059-015-0849-0. 26714481 PMC4699355

[49] Walker BJ, Abeel T, Shea T, Priest M, Abouelliel A, Sakthikumar S, Cuomo CA, Zeng Q, Wortman J, Young SK, Earl AM. 2014. Pilon: an integrated tool for comprehensive microbial variant detection and genome assembly improvement. PLoS One 9:e112963. doi:10.1371/journal.pone.0112963. 25409509 PMC4237348

[50] Gardner SN, Slezak T, Hall BG. 2015. kSNP3.0: SNP detection and phylogenetic analysis of genomes without genome alignment or reference genome. Bioinformatics 31:2877–2878. doi:10.1093/bioinformatics/btv271. 25913206

[51] Letunic I, Bork P. 2019. Interactive Tree Of Life (iTOL) v4: recent updates and new developments. Nucleic Acids Res 47:W256–W259. doi:10.1093/nar/gkz239. 30931475 PMC6602468

[52] Ozer EA. 2018. ClustAGE: a tool for clustering and distribution analysis of bacterial accessory genomic elements. BMC Bioinformatics 19:150. doi:10.1186/s12859-018-2154-x. 29678129 PMC5910555

[53] Seemann T. 2014. Prokka: rapid prokaryotic genome annotation. Bioinformatics 30:2068–2069. doi:10.1093/bioinformatics/btu153. 24642063

[54] Lê S, Josse J, Husson F. 2008. FactoMineR: an R package for multivariate analysis. J Stat Softw 25:18.

[55] Li H. 2013. Aligning sequence reads, clone sequences and assembly contigs with BWA-MEM. arXiv 1303.3997 [q-bio.GN]. https://arxiv.org/abs/1303.3997.

[56] Kokot M, Dlugosz M, Deorowicz S. 2017. KMC 3: counting and manipulating k-mer statistics. Bioinformatics 33:2759–2761. doi:10.1093/bioinformatics/btx304. 28472236

[57] Pedregosa F, Varoquaux G, Gramfort A, Michel V, Thirion B, Grisel O, Blondel M, Prettenhofer P, Weiss R, Dubourg V, Vanderplas J, Passos A, Cournapeau D, Brucher M, Perrot M, Duchesnay E. 2011. Scikit-learn: machine learning in Python. J Mach Learn Res 12:2825–2830.

[58] Wickham H, Averick M, Bryan J, Chang W, McGowan L, François R, Grolemund G, Hayes A, Henry L, Hester J, Kuhn M, Pedersen T, Miller E, Bache S, Müller K, Ooms J, Robinson D, Seidel D, Spinu V, Takahashi K, Vaughan D, Wilke C, Woo K, Yutani H. 2019. Welcome to the Tidyverse. J Open Source Softw 4:1686. doi:10.21105/joss.01686.

[59] Winsor GL, Griffiths EJ, Lo R, Dhillon BK, Shay JA, Brinkman FSL. 2016. Enhanced annotations and features for comparing thousands of *Pseudomonas* genomes in the *Pseudomonas* genome database. Nucleic Acids Res 44:D646–D653. doi:10.1093/nar/gkv1227. 26578582 PMC4702867

