# Erratum for Pincus et al., “A Genome-Based Model to Predict the Virulence of Pseudomonas aeruginosa Isolates”

**DOI:** 10.1128/mBio.00037-21

**Published:** 2021-02-23

**Authors:** Nathan B. Pincus, Egon A. Ozer, Jonathan P. Allen, Marcus Nguyen, James J. Davis, Deborah R. Winter, Chih-Hsien Chuang, Cheng-Hsun Chiu, Laura Zamorano, Antonio Oliver, Alan R. Hauser

**Affiliations:** a Department of Microbiology-Immunology, Northwestern University Feinberg School of Medicine, Chicago, Illinois, USA; b Department of Medicine, Division of Infectious Diseases, Northwestern University Feinberg School of Medicine, Chicago, Illinois, USA; c University of Chicago Consortium for Advanced Science and Engineering, University of Chicago, Chicago, Illinois, USA; d Division of Data Science and Learning, Argonne National Laboratory, Argonne, Illinois, USA; e Northwestern-Argonne Institute of Science and Engineering, Evanston, Illinois, USA; f Department of Medicine, Division of Rheumatology, Northwestern University Feinberg School of Medicine, Chicago, Illinois, USA; g School of Medicine, College of Medicine, Fu-Jen Catholic University, New Taipei, Taiwan; h Molecular Infectious Disease Research Center, Chang Gung Memorial Hospital, Chang Gung University, Taoyuan, Taiwan; i Servicio de Microbiología y Unidad de Investigación, Hospital Universitari Son Espases, Institut d’Investigació Sanitaria Illes Balears, Palma de Mallorca, Spain

## ERRATUM

Volume 11, no. 4, e01527-20, 2020, https://doi.org/10.1128/mBio.01527-20. The files for Fig. S1 and S2 in the supplemental material were incorrect (they were reversed). The files have been corrected online.

