# Supplementary figures and images for: A Genome-Based Model to Predict the Virulence of Pseudomonas aeruginosa Isolates
Source: mBio. 2020 Aug 25;11(4):e01527-20. doi: 10.1128/mBio.01527-20 (PMC7448275; doi:10.1128/mBio.01527-20)

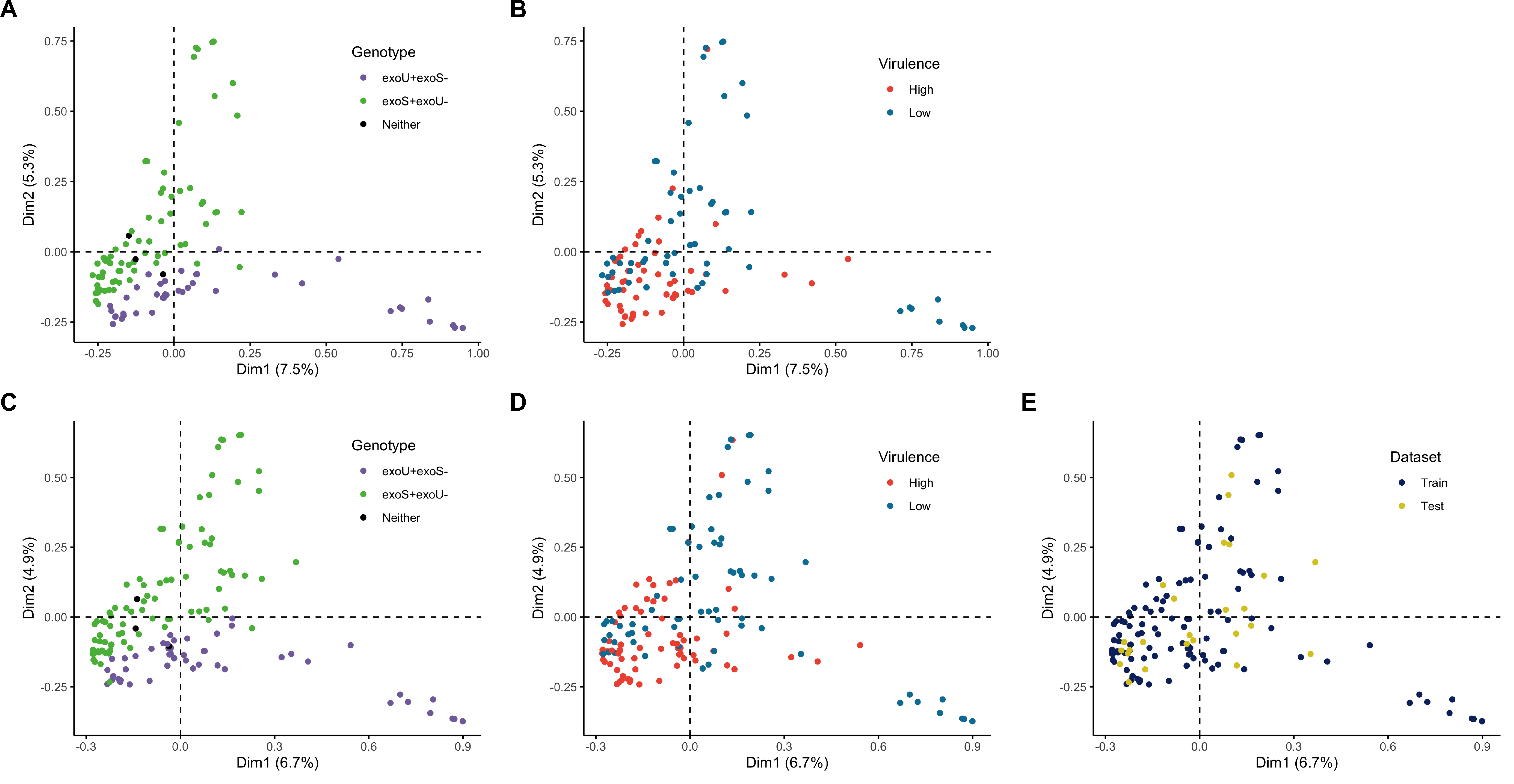

Supplement: FIG S1 [file mBio.01527-20-sf002.tif]

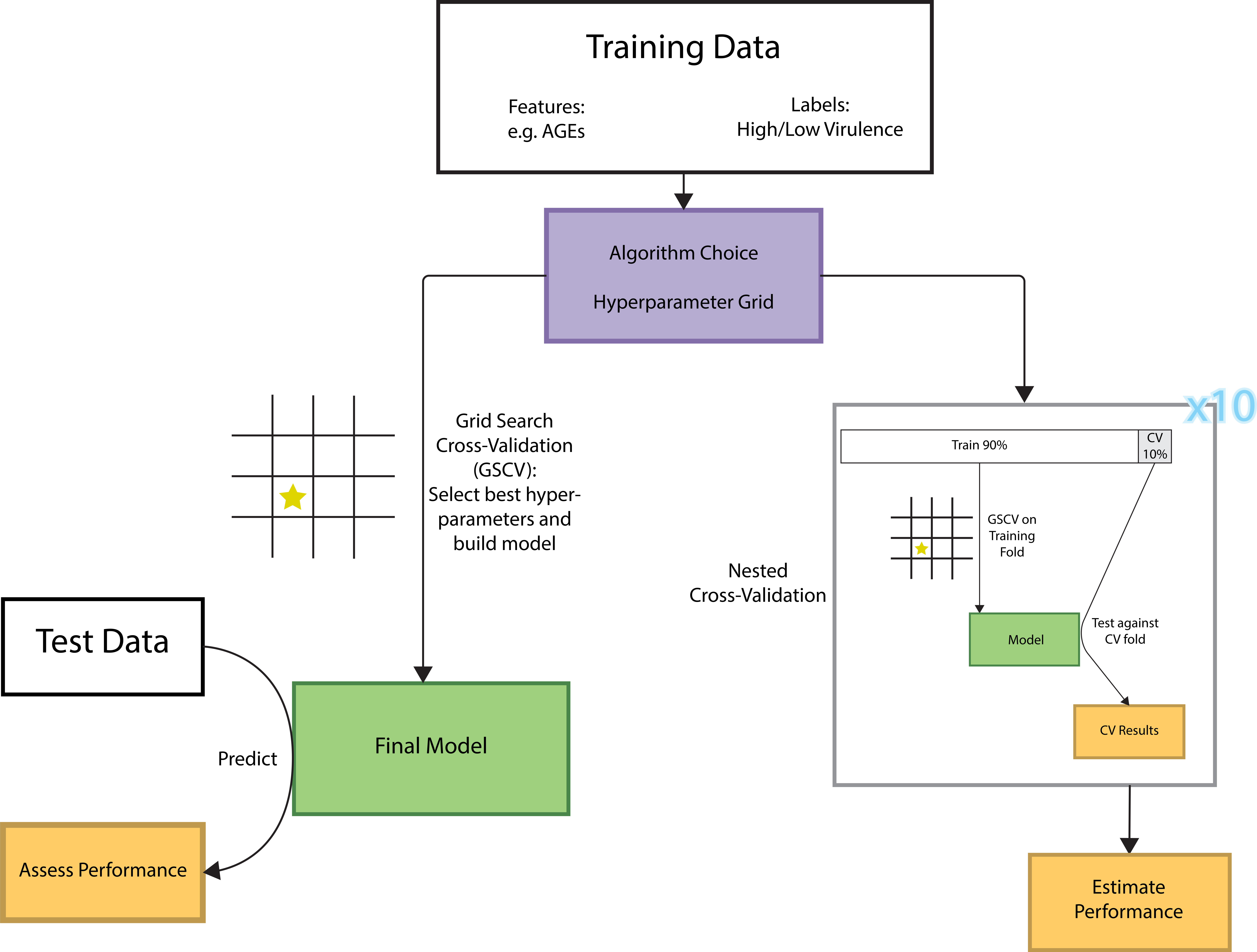

Supplement: FIG S2 [file mBio.01527-20-sf001.tif]

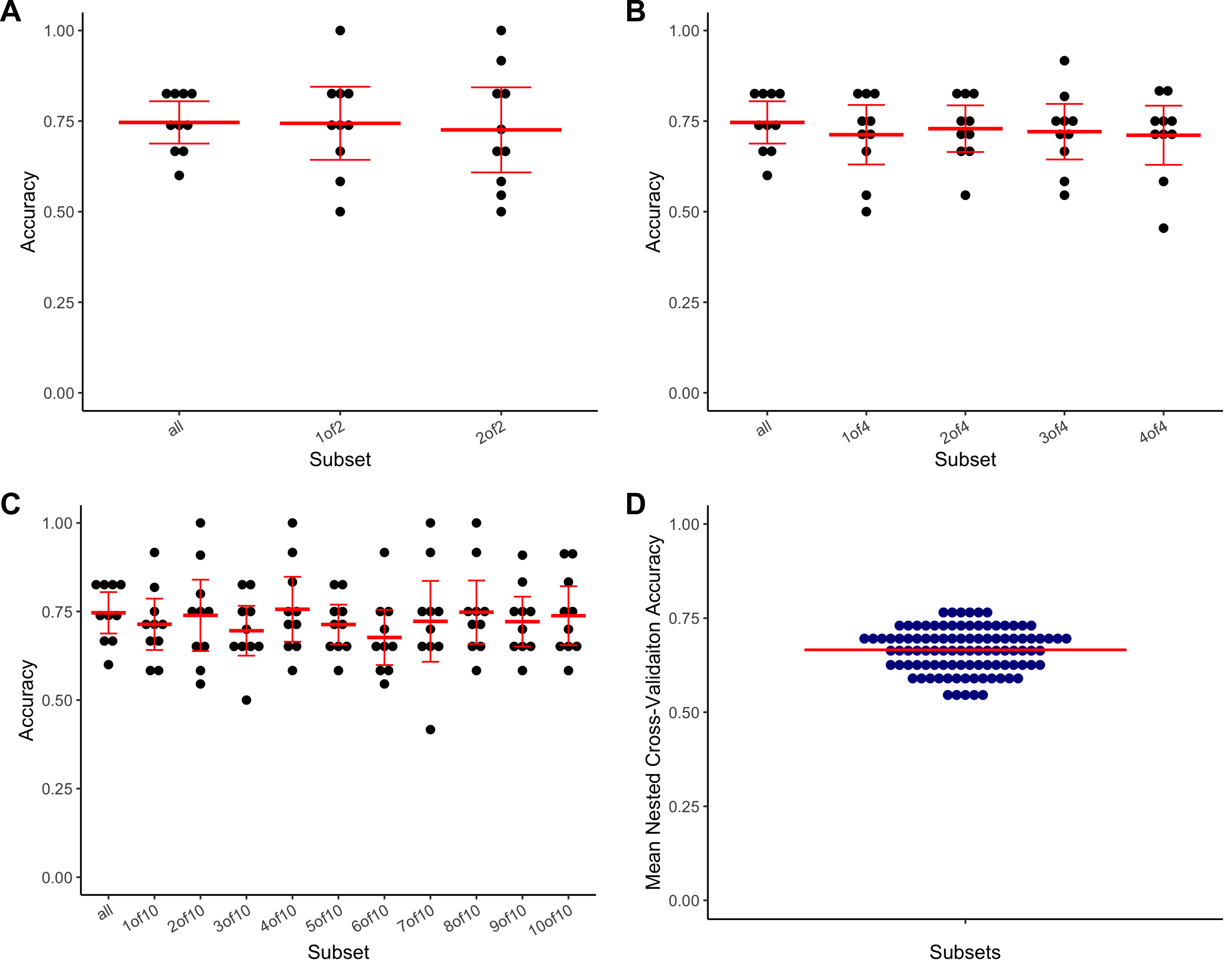

Supplement: FIG S3 [file mBio.01527-20-sf003.tif]

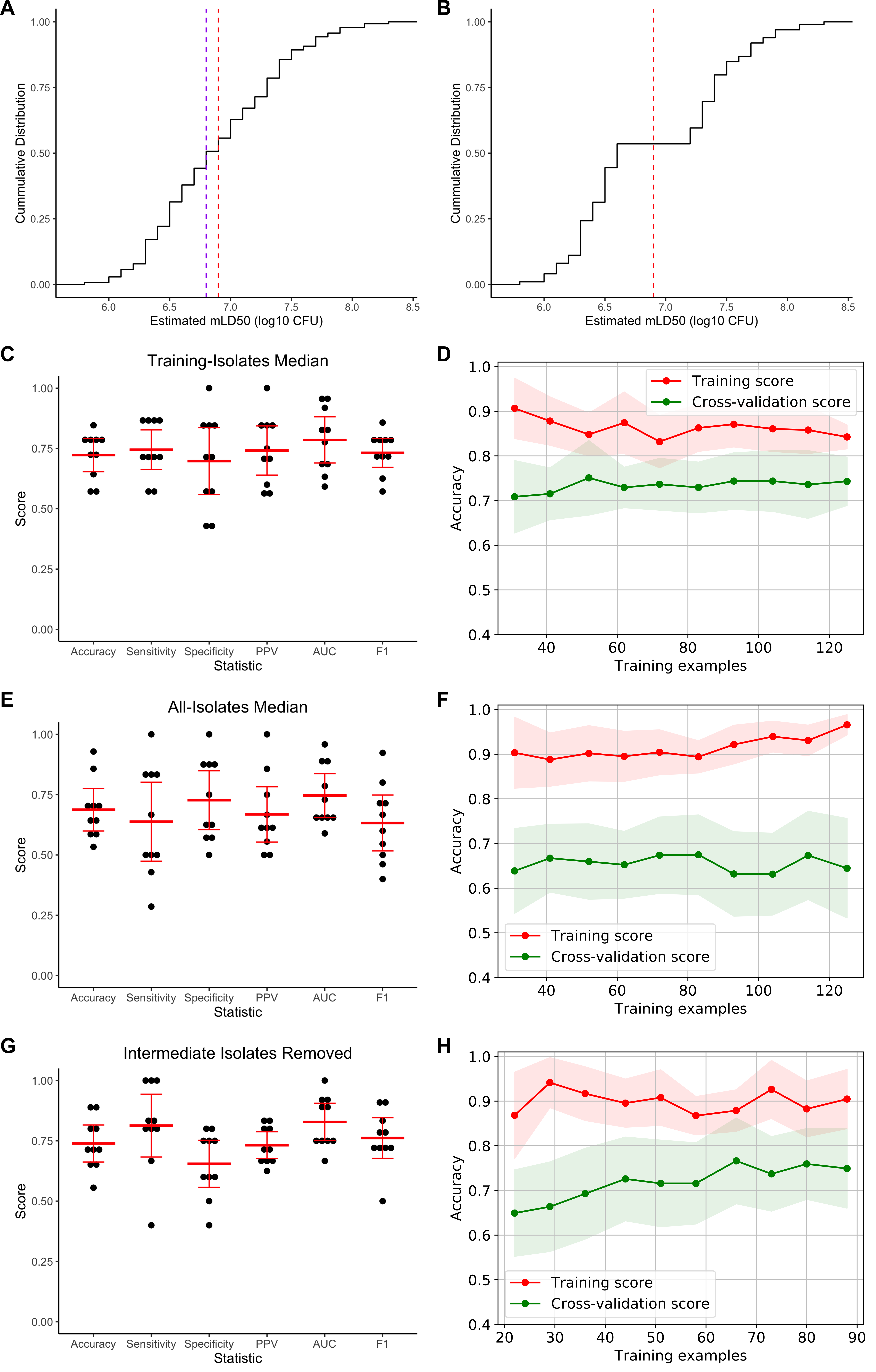

Supplement: FIG S4 [file mBio.01527-20-sf004.tif]
