# Supplementary material for: A Genome-Based Model to Predict the Virulence of Pseudomonas aeruginosa Isolates
Source: mBio. 2020 Aug 25;11(4):e01527-20. doi: 10.1128/mBio.01527-20 (PMC7448275; doi:10.1128/mBio.01527-20)
Supplement: DATA SET S1 [file mBio.01527-20-sd001.docx]

**A Genome-based Model to Predict the Virulence of *Pseudomonas aeruginosa* Isolates**

**Supplementary Data File 1.** Sequences of subelements >50 bp making up the 10 AGEs most predictive of virulence class in the random forest model trained on AGE content of the 115 training isolates

>unique_grp_5582 | bin364_se00006

gccgtcgagcaccgggtcatagttacgggcctgggagaggtactgctccagcgccaccacgcgcttctcgcgcccaatcatgttgcgcgggatgttcttctcgttgagcttggcctcgatctggacgatcacctcccaggccttgggctcggtcttggcgaagtagtgctgggcgtactcgatgaacagcgcgtcgatgttgatgacgtggcgctggatcagcatgtagtccgggcgctggcccagttccaccagggcgcgggcgatgatattgacgaagcgccacgcaaactcgcgaaatgccgcgctgttgccttccccggagagctgccctgcaacacgggtggccacctcgctgatgcgcccaaagcggcccacagcgttgtagcgcgcggaaatgtcgggccagcccaaatggaagacatagaactca

>unique_grp_6841 | bin610_se00004

gcgtcgatcgccaccgtaccggcaaaaggtgctggccgctcgccggattcccagatcagcgccggcgccagtcgcaacaaggtgaatgaatgggaccagcccgtcgtgctgatcacggtcgcgcgccagactgccttcccgtccggcgtgggcttcaacatgccgtgatcctgcagcacgttgaacacggcggtgttgttcgcagggatgccgtcgatgccctgggacagcaggtgtgcgcgcagcttgtccgagaccgtcttgctcaccagccacaagccgtcttcggtgagccagccatcggaggcctcgggttggttcagcttcagctcctccttgagcaggtaacgcaacccgtccagcagcttgcgttgcagtgcgtgcttgggcgcgaccatggcgcgggcaggatcgccgcccagttccagggcgaccgaagcgcgatcggcctgcacgacgagttcgccaagcaccccagcgtgttcgtattggccggcaagaacataaagcagcggtccccagagcgcggggtagccgctgagccagtccaggatttggcgatcgagcagttggtggtggagcaatcccgtcgcggcgctgtgcaggcggtactcgcgatcatcgcggtagcggaagcggtacggctgctgcaacgggccgtgccaagggtgccaagtgctgccgtcggccagttcgacatgcagatcgacggcgatcttgccgatgtcatgcagcagcgcagcgtaggcgacggcggcggtccaggcctcggactgcgccgcttggtcttcggggttggcgccgataggtagcagatgggactgccgtagcttcaggctgtaggcgacgatctccaggccatggtcgagcatgccgccggggtacgcatggtgatgggcctc

>unique_grp_1425 | bin20_se00056

ccagcacgacccacgaccggacgctgattgcgccgacgcactctgcttcttcatgtgcgttgccaggtcgcactgggttcggatgtctgacgaatggctgtgggaccgatggccacgtcgcgcctgaatgtcgtcctcccgtgccaagtagcacttccttgtccgggcgttcagcgcgaggcgtcaaccattttcccgcgctggagaggggatttgccaggcgaaagcatccgcgcaatcgccaactgaatcgcccgtctgcgcgcctcgtgctcctgcgaatccctcgccgccggtgccatgcaccacgtttgcacgatcgtcgctaccagtcgcacgatctcatcaggcgtgaaatccgctgtgatgcgtccttccgcctgggcagcggtgatggccgcatgtttggcttccagtacgctgtgaccggcggggagcaggagcacttcttggcctcgctccaggtggaaccaggtcatgagccgccataggtgcggccgctcggtgagcaaatcgtaggtgcgccccgcatagccgggaagatcgtccgcatcgaagcgctcgtcctgcgccgcggcgaggatttcccggctgagcacggcgtcgaagagagcctccttgtccccgaaatggacataaagcatcggcttgctgatgcctgctgccgccgcgatccggtcaattcgtgcccccgccagcccgttctcggcaaattcccccagcgcagcgtccagcagcatttgctgggtctcggcgggacgtctcttggccatgggccgttactcctcaacaacatttcatccgagcgcccatcatcgcttgacacgggcgccattaacttacaaactagttggtaagtaaaccacaggagtgcatttatgtccagacattggttcctcaccggcgcatccagcggcatcggtcgccatcttgctgaaatcatcctggcttcgggggatgacctcacggccaccgtcaggcgtcccgagacgctcgacgacctggcagcacgctacggtgaccagctcgttgtcgaacggctcgacgtgaccgtgaagggggacatcgcacccgtggtccagcgcgccgaggcccgccgcccggtcgatgtcctggtcaacaacgccggcggcggcatcatcggcgccaccgaagagttttccgacgcggacatcgaagggcagatcagcctcaacctgctggcgccggtgcacgtcacgcgggccttcattcccgccatgcgcacacggcgagccggtcggatcatccaaatctccagcgccagtggccaaggctccctgccgaccagcagtctttaccacgccgcgaaatggggtctggaaggcttcagcgaatgccttcgccaggaactggaggggtttggggtcttcgtcacactcattgaaccgggcggcgctcgcacgagcttcagccgcaatttgcagtacgcgccggcgaaccccgcctatcaggacacgcccgctggacagattcgcgcgatgttcgagaacgcgggtgacgagttgtacacgctcgatccgcagaagatcgcgcaccggatcttcgacacggccacgagcgagaagccaccgcttcgggtggcgctgggcggtgacgccttcggcgtcatccaggcggcgctcaagggcaggctgtctgcccttgaagcgcaggagagtctggcgcgatcggtggccttcgatagctgatcgctgtt

>unique_grp_6842 | bin610_se00005

cgatgcagggaactgctggaccaactcggcgtagcactccagcggagcgcgatagaggatggcgaactgcttgcgcgacagcgatgtgcgctgccagatgtgttccagcagcttttgccggcgcggtatcgccagtagggatgcggccgactcgggccgcagcagccctttcggaagatcagtggctggcgttggcgacggagcggcagcgaccgaggcccgttttcgctggaacagggagagcatgagggtgtcctgatggcgggccgaccgggaggcctttttgctttttcgaggtagggcctttccccttgcactccattcccttcccctttcggccttttggcccttaacagcctttgggtatag

>unique_grp_6989 | bin654_se00007

cgtcatctcactgaaatgtctgaattcctaggattaaatctcctactgagggagatttaaaaaatgaagttattcagaaattacatcatcgcactagcaattatcgctcttgctcccgcctttgctgacggctcgacggatcgactgtatggtgaaacgctgcaagcaaatgaagcttccacacgtgcatatgccttgaacgcaggaaagaatcctgcctcccgtcgtccattatcgctacggaatgacgcttgatatcgctaaggtcatcgggctcacgcccacctatggaaagtgcggaataattccggcgcagatgtcttatgtggaatcgaaaggagaaatcaacatactcgaatatcgcgcttcggggagcggctgtcgaaacgagaactgactgtacgagatgcgatttacagtattcaacttgccctag

>unique_grp_1437 | bin20_se00073

ttgcgcgctgtgaggaacagacgagacccaaggaaagcgaacgaggcctgtcgcacttcgttggtaatggtcgtgcggactgttcgctgcacaaaaatgccgaatgaattttcagcaatctcacggccgacttagacgacgtgaactgcggcggtcggcgtagcggatacatcgccctacgtcgaccctgccggcgcgatccactcagtcgactgtcaatctcacaagtcccgcctattctcgctcaaggatacgcgcaccgggcccctggcctgcgagtatgtcgcccggattacgcaacgggcactctttgagagagagacaaccgcacccgatacatcccccgagttcgtcccgcagccgcatcaactgcgtaatgcggtcgtcaaggtcttcacgccacctagacgccacacggcgccaatgcgcagcagtcagatgaacgcccggctgatactgcccgagagcgtcgagcacttcgtccagcgatagtccggcccgctgcgcgactttgacgatggccagatacctcaagatagcgggcg

>unique_grp_1437 | bin20_se00075

tcatgcgaggctcgcaacctagccttgtcagtcttgcgggtgagttggcctatggagcgattgtttccgcggatcatcctggcgcttcggtgcgggaaatggttttgctcagcgccgcaaggcagactgccttacgtggcgggcgaggacgtggcgcgcaccgctgcggcggtactgcgggcggaccctgttccgcccggagagctggcgatcaccggcccgcaggctctcaccgccgaggcgctggtgaacagcatcaatatcatcttcggagccagcatcgacctggtgcccgtcagtgaggaagccctggcgctgcatctgcaggtctcgggctttccgaaatccacggtgcgagaggcgctgatcatcgaagaggtgtccaaacgcgggctggcgccgttctccgacggtgtcatcgagcagatgaccggtcagccgccgcgcagcatcgaggcggtgctggtcgaacaccgcctcgatttgctgttgtccaccagcaccccccggctttgagacgagtcgacttcttcgactctagaggctgcaggcgtgcttgctgcagtacatgatccacgccgagacgatgatgaaacaacgatctgtacgaacagccaccccgacgacggtggtcgccaagccttttgccacggaacgtgtgccaattgttggcgacgtcgccagcctcggcggcgtaaccgtgtccaacgttcgtttctatgaagcagagaggttgatccatgctacgcgcacggcaggcaaccagtgcctctatgggcccgtatctagcacctatgccagtttcaagtagggattgttcggtggccgctcatcgaacagttggccagggtcatcgagtaagtagccatgcagcttgcgcgacttgcgcgggccggtgacctcgcaggtccagatgtttaagctgctgggctgcttgcgatggacctgcagcttctcgaagcgcttctggacccattgccagtcgacaacggcatcctgcttggcgagtgctggaacttgaggatgctcctggacatagcgctgaaacaccccagggcttaccaggaacaccgtgtcgtccacggtatgcaccagtgccttggcatcgttgaggatcagctttcggctctgcaagccatgcctaagccagcccatgaagtgctcgcccgaaggctgctgtgtcgatgccgaagatttagacgctggcacaggcagtaccagcgaactcgttgcgaaggccgggacgggctctgcatcctgtcgcgtcgcaaggaggtgtccggagcccaccatcgccagcatgtcttccatgacgtcaggcaggggctgaactgcgggcggtgaagccaagttagcggggctgctgccttcccaaggtggcgtcgtctgttcttccaagacaggcttcgcggcagtcataggcggggcagcctccgagccagcatccttctcgattgccgcagc

>unique_grp_8120 | bin987_se00001

gatgcctcttggaggttacgaagtggtcgcgcggcgttcgtaccagccgcgtgagcgattgaccacgcgcaccaccagcagcatgaccggcacctcgatgagcacgccgaccactgttgcgagtgctgcgccggaatggaagccgaacaggctgatggcggcggccacggccagctcgaagaagttgctggcaccaatcaacgcggatggcccagcgacgcagtgcttttcgcccacctggcggttgagccagtacgccagaccggaattgaagaacacctggatcaggatcggcaccgccagcatggcgatcaccagcggctgccggatgatggcctcgccctggaaggcgaacagcagcaccagcgtcagcaacagcgcggcgatggacaatgggccgatgcgttccagtgccctgtcgaaaacggcctgccccttgcgcagcagcgcccggcgccagagctgggccaggatgaccggaatgacgatgtacaaacccaccgacaccagcaaggtgtcccacggcaccgtgatcgacgacaggcccagcagcaggccgacgatgggtgcgaaggcgaacaccatgatggtgtcgttcaaggccacttgcgacagcgtaaataccggatcaccgccagtcagccgactccaaacgaaaaccattgcggtacacggtgcagcagccagcaggatcaggccggcgatatagctgtcgagctgatcggcgggcagccagtcggcaaagacgtggcggatgaagatccacgccagcaacgccattgagaacggcttgacggcccaattgatgaacagcgtgacccccatgccccgccaatgctggcgcacctggccaagcgcggcgaagtccaccttcagcagcatggggatgatcattacccagatcagcaggccgaccggcaggttgaccttggcaatctccatgcggccgatggcctggaatgcggcgggcgcgaactggcccagagcgatccccgcgatgatgcacaccgccacccatagcgtcaggtaacgctcaaagccgctcatggcggagtttgctggcgcggggccggcagtatcggttgcgctcatcgggcgggcctcactggcggccgatgtcgcgcagttcacgctgcaacgacatggcatcgagactcttgatcggaagcgacaggaacaactcgatacgtcggcgcagcgtcatggcggtatcgaagaacgccttgctttgtcgttccagggtgccttccacggctgccgggtcaggcacgccccaatgcgccgacaccggcttgcccggccacagcggacacgcctcgccggctgcgttgtcgcagacggtgaagatgaaatcgaacaccggcgcatcgggcttcacgaactcgtcccagctcttgctgcggtagcccgttgtcggaaggtgcagccgctccagcgtggcgagtgccagcgggtgaacttcgcctttcggatggctacctgccgaccaggcgtgaaaccgcccctggcccatgtcgttgaggatgccttcggcgaggatggagcgggccgaattgcccgtacagatgaacaaggcgttgtaagtggtatcggtcatggtcagcagcaggtcttggatggagaaacctcgcatacgccaccttggcagcaatgctcggtcaggtagccgatgaggccgttcatgtggccgtactcggcccgatagatcaggttgcgaccctgctgctcgatggtgacgaggccggcatgggccagctccttcaagtggaaggacaaggtgttgcgggccacgtcgagctggtcagctagcgtgctcggcgtcagtccgcccgggccagcaacgaccaaggcgcggaacacgcgcaggcgctgggtgtgagccagggcgtttaaggcggaaatggcttgatcctctttcatgattcgataatacaacatttatagaattaatgtgcaaaccacagttggatttgcgggttgcgctatgtgttggactgcaaaagcgggcaccccccagttcgcatttctgactgaccgcgcagccgcgcgcagccaaggtatcggcatcgcaacagaagagccgatgccatgcctgcaatccatgtatcccggtgtctggtcggtaccagcctcctggccgcagcc

>unique_grp_8120 | bin1807_se00001

tgctgggcctgatccgcaacagcggcgaggaaccgaccatcatcgagtacctgaagacgccgcccggccgcgacacgctccaggcgctgatcgcggcgatgggcgtgccggtgcgggacatactgcgcgagaaaggtacgccctatgccgaactcgacctgggcaacccgaagtggagcgacgacgacctgatcggtttcatgctccagcatcccattctcatcaaccggccgatcgtggtcacgccgctgggtgtgcgcctgtgccggccatcggaagccgtgctcgaccttctgccgcaaccgcagcgcggcgcgttcaacaaggaagacggcgagccgctggttgatgagaaaggccgtcgtgtctgaagcccgcctcgacctgccaaacatcgacgcggcgctgttccagcaaccagacgccgaacacctgttcgcgccggcacggaccacccacgcgccgcgcttcctgctgctctacggctcgctgcgcgaacgctcgttcagccgcctcgcagccgaagaagccgcccgcatcctgcgggcgctgggcggtgaaacccgattgttcaatccgtccggcctgccgctggtggacgatgcacccgctgatcaccccaaggtcaaggaactgcacgaactggtgcaatgggccgaaggcatggtgtggagtt

>unique_grp_1423 | bin20_se00057

tcaggtcaagcccagggcgacggcaaagccagcagcagccatggccgcgtacgacgggagcactctccatgcgagcgccctaccgcccagcgacatcaacaggacaagtttggtgacctgattgacggatgcggcgatgaacaaggccagggtggccgaagcggcggtgacggcgccctcggtcaccaggcggccaatcgagagcgtgatcgcatcgacgtccaccagacccgaggcagcggcggtcagcatgacgccagcatccccgaagcgatcggcaagaaagcggcctgccagcatcaccgcggccaggaaggctgcgaactggagcgcggagcgcagctgcaatgggttgccaagctgaagttcgggcaagtcgtcggcgggcgaccgcgccgccaggaacgtccccagaacaccgccagccacggccatggccacgaagatgaccaccgggtcccagccgatgccgggggctgcgatggcgacgagagccgccatgcgcgcaaacatggctgcgcaggcgagcgccgcccccgccgctgccagtggatgccagcctggcgtctgcccggcccagcgcgccaacgtggccgtggtggcggtgctcgacacgatcccgccgagcagcgctgtgaataccagccctcgtttcctgccgagcgagcgcatcaggacgaacccagcgaacgacagcactgccagcaggacaacggcccaccaaaggcgatagggattcaacgcctcataggggccaaagccacgatcagggagtacgggcagtacgaccacgctgatcaacagcaactgcgtgccgctgctgatctccgcctcggacagcttgccgacgccggcgtgcaagagctgcttgaaacgcagcagcgccagcacgaccacggcggcgaccgccgaaacgcgccaagcgccactggtcgccagcgccccgatggcgaaagtcgcaagggcggcgacggacgtggtcagtcccatcacggcatgcatatgcgccgtcaccaggtagcccaccaccagatacaccccgacgagtacgagcagcgcgatgagcatgccggctccccagcgctgggccagcagcccgccaaggccgccgagcaggccgatcagcgcaaaggtgcgaacgcccgcgacgcgatgtccctcgggccgctcgcgttgctgccatccccgttccaggccgatcagaagtcccgaagcgagtgccagcccgagcccgaccagcgcgggatcatcgaattcactcatcgtcgaa

>unique_grp_1435 | bin20_se00069

aaggcggccgagggcgttgcgcagcactccgcccagcatcagggccgcgacgaacaggaagggccaggcctgcccgatgtcgttggtctgcccctgccgccgtgggtccgccgcaggcagggcctcgccatcgatcacccgcatgatctgttcggcgcctgcggcgatgccgccgtcgaaatcgccctggcgcaggcgcggcgtgatcacctcgtcgatgatgcgccgcgataccacgtcgctcagcgcaccctcgatgccgtatccgacttcgatgcgcacggtgcggtcgtccttggccaccaggagcaacgcgccgtcgtccaccttgcgacggccgagcttccattgctctaccacccgcagggtgtactgctcgacgggttcgggccgcgtcgagcggaccatcagcacggcgatctgcacgcccttgcgttgctcgaaggcccgaagctgcgcatccagcgcggcgacacgatcggcggcgagcgttcccgtgagatcggtga

>unique_grp_5112| bin258_se00005

ttgcgctcgctatacctgtccaccagatacggcgacgcatcgcgcgtgagcagcgtgaacttcatcagttcctccatcacgtccacaatgcggtcgtagtaggccgacggtttcatgcgtcccgcttcatcgaattcttgatacgccttggcgaccgaggactggttggggacggtcagcatccgcatccagcggcccagcacgcgcatctgattgacagcattgaaagattgcgagccgcccgacacctgcatcactgccagcgtcttgccctgagtcggacgcaccgcgccgactgacagcggaatccagtcgatctgcgttttcatcaggccggacatcgcgccatggcgctccggcgaactccacaccataccctcggcccactgcgcgagttcgcgcagttccctgaccttggg
